# Genome-wide identification and characterization of long non-coding RNAs involved in the early somatic embryogenesis in *Dimocarpus longan* Lour

**DOI:** 10.1186/s12864-018-5158-z

**Published:** 2018-11-06

**Authors:** Yan Chen, Xue Li, Liyao Su, Xu Chen, Shuting Zhang, Xiaoping Xu, Zihao Zhang, Yukun Chen, Xu XuHan, Yuling Lin, Zhongxiong Lai

**Affiliations:** 1 Institute of Horticultural Biotechnology, Fujian Agriculture and Forestry University, Fuzhou, 350002 Fujian China; 2 Institut de la Recherche Interdisciplinaire de Toulouse, IRIT-ARI, 31300 Toulouse, France

**Keywords:** *Dimocarpus longan*, Early somatic embryogenesis, lncRNAs, Illumina HiSeq sequencing, qPCR

## Abstract

**Background:**

Long non-coding RNAs (lncRNAs) are involved in variable cleavage, transcriptional interference, regulation of DNA methylation and protein modification. However, the regulation of lncRNAs in plant somatic embryos remains unclear. The longan (*Dimocarpus longan*) somatic embryogenesis (SE) system is a good system for research on longan embryo development.

**Results:**

In this study, 7643 lncRNAs obtained during early SE in *D. longan* were identified by high-throughput sequencing, among which 6005 lncRNAs were expressed. Of the expressed lncRNAs, 4790 were found in all samples and 160 were specifically expressed in embryogenic callus (EC), 154 in incomplete embryogenic compact structures (ICpECs), and 376 in globular embryos (GEs). We annotated the 6005 expressed lncRNAs, and 1404 lncRNAs belonged to 506 noncoding RNA (ncRNA) families and 4682 lncRNAs were predicted to target protein-coding genes. The target genes included 5051 *cis*-regulated target genes (5712 pairs) and 1605 *trans*-regulated target genes (3618 pairs). KEGG analysis revealed that most of the differentially expressed target genes (mRNAs) of the lncRNAs were enriched in the “plant-pathogen interaction” and “plant hormone signaling” pathways during early longan SE. Real-time quantitative PCR confirmed that 20 selected lncRNAs showed significant differences in expression and that five lncRNAs were related to auxin response factors. Compared with the FPKM expression trends, 16 lncRNA expression trends were the same in qPCR. In lncRNA-miRNA-mRNA relationship prediction, 40 lncRNAs were predicted to function as eTMs for 15 miRNAs and 7 lncRNAs were identified as potential miRNA precursors. In addition, we verified the lncRNA-miRNA-mRNA regulatory relationships by transient expression of miRNAs (miR172a, miR159a.1 and miR398a).

**Conclusion:**

Analyses of lncRNAs during early longan SE showed that differentially expressed lncRNAs were involved in expression regulation at each SE stage, and may form a regulatory network with miRNAs and mRNAs. These findings provide new insights into lncRNAs and lay a foundation for future functional analysis of lncRNAs during early longan SE.

**Electronic supplementary material:**

The online version of this article (10.1186/s12864-018-5158-z) contains supplementary material, which is available to authorized users.

## Background

The long noncoding RNAs (lncRNAs), which are noncoding RNAs over 200 nt in length, are devoid of open reading frames (ORFs) and are mainly transcribed by RNA polymerase II (Pol II). lncRNAs are spliced and have a cap structure with a poly A tail, lncRNAs have lower expression levels of sequence conservation than protein-coding mRNAs [1]. lncRNAs come from the intergenic, intronic or coding gene regions in the sense and antisense directions. According to their genomic transcriptional positions, lncRNAs can be grouped into three classes: antisense, intronic, and intergenic. It has been reported that lncRNAs play important regulatory roles at the gene transcriptional [2], post-transcriptional [3], translational [4] and epigenetic [5, 6] levels, and are involved in variable cleavage, transcriptional interference, regulation of DNA methylation and protein modification. Interactions between lncRNAs and microRNAs (miRNAs) are also widespread. Cai et al. (2007) identified an endogenously expressed 23-nt miRNA derived from lncRNA H19 in human keratinocytes and neonatal mice, and demonstrated that H19 could be used as a primary miRNA precursor [7]. Moreover, some lncRNAs have miRNA-binding sites and can function as endogenous target mimics (eTMs) of miRNAs [8]. In regard to embryogenesis, some lncRNAs identified in mice were found to not only be controlled by embryonic stem cell transcription factors but also regulated by the mice embryonic development status [9]. In plants, lncRNAs have been found to regulate growth and development [10], reproductive development [11] and stress responses [12]. However, while there have been a few reports on the regulation of lncRNAs in animal embryos, studies on lncRNAs in plant somatic embryogenesis (SE) have not been reported to date.

Longan (*Dimocarpus longan* Lour.) is an important fruit tree that originated in southern China and Vietnam, and has a wealth of pharmacological uses such as in antioxidants, hypoglycemic drugs and nervous system regulators [13] besides supplying fruit. Longan embryo development largely influences seed size, fruit set, fruit quality and yield. In-depth embryological studies of longan are of great significance for the longan industry [14]. A longan SE system established by Lai et al. [15] is considered an excellent model systems for woody plants SE. Recently, our laboratory has conducted systematic research on non-coding RNAs during early longan SE, mainly focusing on miRNAs. A total of 643 conserved and 29 novel miRNAs (including star strands) of 169 miRNA families were identified during longan SE [16]. The molecular mechanisms of miR167 [17], miR390 [18] and miR160 [19] in the regulation of the auxin response factors were demonstrated in longan SE. In addition, miR159 family members [20] and miR171 [21], which are involved in flower organ formation, were found to have the most stable expression among miRNAs at different developmental stages of longan SE [22]. In 2013, our laboratory constructed a cDNA library from a longan friable-embryogenic callus RNA sample and a large number of specific genes were identified [23]. All of these studies were focused on miRNAs and mRNAs in longan. Now, however, with the release of longan genome data from our laboratory [24], investigation of the regulatory roles of lncRNAs in longan SE has become possible. Therefore, based on the longan SE system and Illumina HiSeq sequencing platform, we isolated and identified lncRNAs from three samples of early longan SE, i.e., embryogenic callus (EC), incomplete embryogenic compact structures (ICpECs), and globular embryos (GEs). We explored the functions of the lncRNAs and their target genes in the three samples and speculated that lncRNAs might participate in the developmental process, and then predicted the regulation of lncRNAs, miRNAs and lncRNAs during early longan SE. Finally, qPCR assays and transient overexpression or inhibition of expression techniques were used to validate key lncRNAs, miRNAs and mRNAs. The results enhance our understanding of the regulation of lncRNAs and provide valuable information for research on lncRNAs during early longan SE.

## Results

### Illumina sequencing and identification of lncRNAs during early SE in longan

To investigate the regulatory roles of lncRNAs during early longan SE, lncRNAs obtained from EC, ICpEC and GE samples of longan were sequenced by Illumina HiSeq sequencing. An average of 195,823,419 original sequences were obtained per sample. From 12.69 Gb data, 45,124 transcripts were detected among the clean reads. We distinguished between mRNAs and lncRNAs based on transcriptional codes. In total, 7643 novel lncRNAs, 16,162 novel mRNAs, and 31,007 known mRNAs were identified (Additional file 1); all lncRNAs identified in our research were novel lncRNAs.

**Fig. 1:**
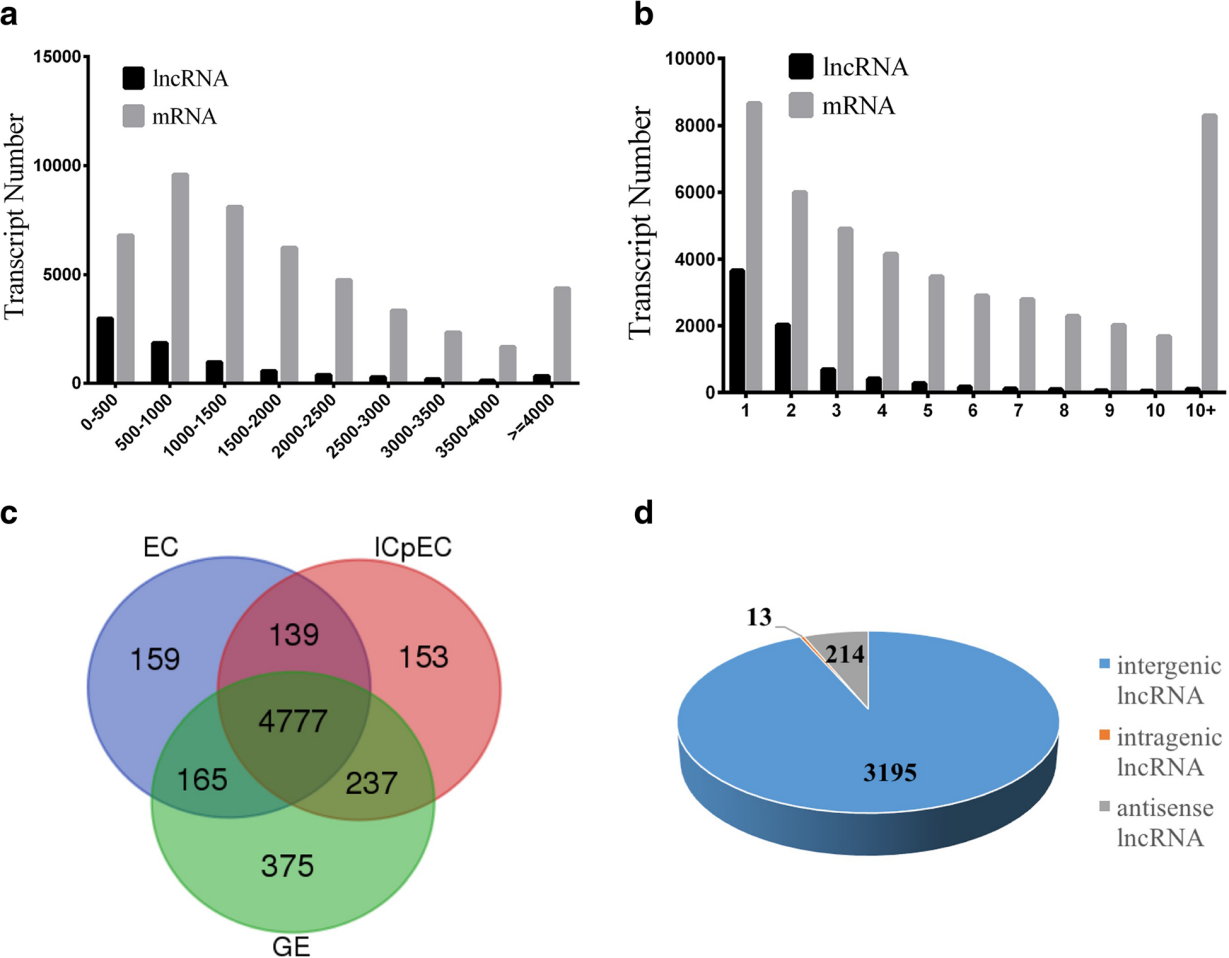
Characterization of lncRNAs during early longan SE. **a** Length distribution; lncRNAs are shorter than protein-coding genes. **b** Exon number; lncRNAs have less exons in longan. **c** Venn diagram showing the specifically expressed lncRNAs in the EC, ICpEC and GE samples. **d** Classification of lncRNAs by genomic location (intergenic, intragenic and antisense)

It has been reported that compared with protein-coding genes, lncRNAs are shorter and have smaller numbers of exons in plants [25]. The length and exon number distributions of the 7643 lncRNAs in all three early stages of SE were compared with those of all 47,169 predicted mRNAs. The results showed that 83.04% of lncRNAs were 0–2000 bp, 12.64% were 2000–4000 bp, and only 4.32% were > 4000 bp in length, By contrast, 7.19% of mRNAs were > 4000 bp, 65.13% were 0–2000 bp, and 25.64% were 2000–4000 bp in length (Fig. 1a). Notably, 74.39% of lncRNAs in the early longan SE samples contained only one or two exons, whereas the number of exons in 82.41% of the mRNAs ranged from 1 to 10. In contrast to protein-coding genes, the lncRNAs had a smaller number of exons (Fig. 1b).

RNA-Seq expression analysis revealed that among the 7643 lncRNAs and 47,169 mRNAs identified, 6005 novel lncRNAs, 15,233 novel mRNAs, and 23,886 known mRNAs were expressed in the three samples. Of the 6005 expressed lncRNAs, 5254 were detected in the EC stage, 5320 in the ICpEC stage, and 5568 in the GE stage. Additionally, 4790 lncRNAs were shared among the three samples and 160 lncRNAs were specifically expressed in EC, 154 in ICpEC, and 376 in GE. The number of lncRNAs and specific lncRNAs identified at the GE stage was the highest (Fig. 1c).

### LncRNA classification and family annotation in longan early SE

According to the locations of the nearest protein coding genes, 3422 of the 6005 expressed lncRNAs were classified into three categories: intergenic lncRNAs without any overlap with protein-coding genes, intragenic lncRNAs positioned in protein-coding loci, and antisense lncRNAs overlapping with exons of a protein-coding transcript on the opposite strand [26, 27]. Of the 3422 lncRNAs, 93.37% were intergenic, 0.38% were intragenic, and 6.25% were antisense (Fig. 1d).

lncRNAs are divided into distinct ncRNA families based on the evolutionarily conserved secondary structure of the RNA, the *cis*-acting elements of mRNA, other RNA elements, and the common ancestors of ncRNAs. We annotated 6005 lncRNAs (Additional file 2), among which 1404 lncRNAs belonged to 506 ncRNA families, with 1766 groups (a lncRNA could be annotated as belonging to multiple families). Of these 1766 groups, 53.63% were annotated as miRNA families, 25.50% as snoRNA families, and 4.99% as sRNA families. Only 5.06% were annotated as lncRNAs families, including the oxidative stress-induced *adapt33* family [28, 29], the *DLX6* family involved in embryonic organogenesis [30] and the methylated *KCNQ1OT1–1* family [31]. The remaining groups were annotated as the virus-associated families *Corona-pk3* [32], *flavi-FSE* [33] and *HIV* [34].

### Differential expression of mRNAs and lncRNAs during longan early SE

**Fig. 2:**
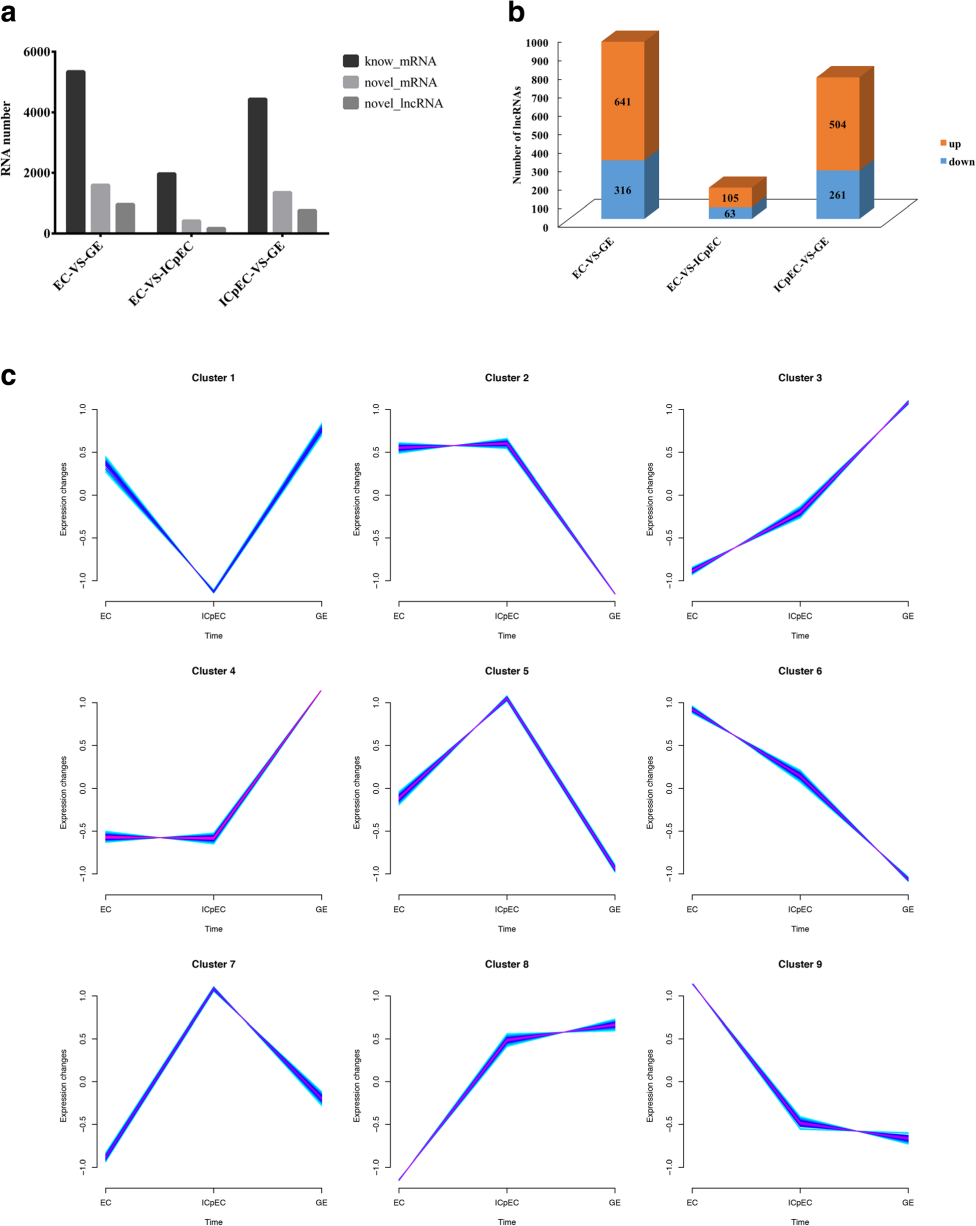
Differential expression of early longan SE-related lncRNAs and mRNAs. **a** Differentially expressed lncRNAs and mRNAs between the EC, ICpEC and GE samples. **b** The number of significantly differentially expressed lncRNAs in EC vs. GE was greater than in EC vs. ICpEC and ICpEC vs. GE; up-regulated (orange) and down-regulated (blue) lncRNAs are shown. **c** Time series analysis of 3680 lncRNAs based on their expression at the gene level

In this study, we detected 39,119 genes encoding proteins associated with longan SE in three samples, and determined the expression levels of these genes by Fragments per Kilobase per Million (FPKM) mapped reads values. Large-scale expression profiling through FPKM values showed that the number of differentially expressed mRNAs was 3877 in EC vs. ICpEC, 7746 in ICpEC vs. GE and 9222 in EC vs. GE. Compared with EC, 5317 mRNAs were up-regulated and 3905 mRNAs were down-regulated in GE, and 2057 mRNAs were up-regulated and 1820 mRNAs were down-regulated in ICpEC. Compared with ICpEC, 4728 mRNAs were up-regulated and 3018 mRNAs were down-regulated (Fig. 2a).

In the significant difference analysis of the 6005 lncRNAs, we found that the number of significantly differentially expressed lncRNAs in EC vs. GE was greater than that in EC vs. ICpEC and ICpEC vs. GE (Fig. 2b). During early longan SE, 1259 and 335 lncRNAs were significantly expressed in EC vs. GE and EC vs. ICpEC, respectively. Compared with EC, 802 of 1259 lncRNAs were up-regulated and the other 457 lncRNAs were down-regulated in GE, and 229 of 335 lncRNAs were up-regulated and the other 106 lncRNAs were down-regulated in ICpEC. There were 1028 significantly differentially expressed lncRNAs in ICpEC vs. GE. Compared with ICpEC, 604 of the 1028 lncRNAs were up-regulated and the other 424 lncRNAs were down-regulated in GE.

The lncRNAs showed different expression trends during longan early SE according to the FPKM values. In this study, the expression of 3680 lncRNAs over time was analyzed based on their expression levels in different SE stages to identify expression trends. As shown in Fig. 2c, the expression trends of the 3680 lncRNAs in the three samples were divided into nine major categories. The expression levels of 273 lncRNAs, which accounted for 7.42% of the total lncRNAs, from the first category were lower in the ICpEC stage than in the EC and GE stages. The lncRNAs in the fifth (386 lncRNAs) and seventh (361 lncRNAs) categories were expressed at higher levels in the ICpEC stage than in the other stages. The lncRNAs in the second and fourth categories showed stable expression from the EC to ICpEC stage. The second category included 369 lncRNAs (9.89% of the total) whose expression decreased in the GE stage. The fourth category contained 688 lncRNAs (18.70% of the total) whose expression increased in the GE stage. The third and sixth categories showed two completely opposite trends: the 477 lncRNAs (12.96% of the total) classified into the third category exhibited higher expression levels in the GE stage, whereas the 340 lncRNAs (9.24% of the total) classified into the sixth category showed higher expression levels in the EC stage. The lncRNAs in the eighth and ninth categories showed stable expression from the ICpEC to the GE stage, but the 417 lncRNAs (11.33% of the total) classified into the eighth category exhibited lower expression levels in the EC stage, while the 373 lncRNAs (10.14% of the total) classified into the ninth category exhibited higher expression levels in the EC stage. The lncRNAs in the second, fourth, and eighth categories were accumulated from the EC to the GE stage, indicating that the related lncRNAs in these three categories contributed to the formation of the GE stage. The expression decrease from the EC to GE stage in the sixth and ninth categories indicated that the lncRNAs in these two categories were mainly involved in the maintenance of the EC stage. The lncRNAs in the second, fifth and seventh categories were highly expressed in the ICpEC stage, and these lncRNAs were possibly involved in ICpEC morphogenesis. The specific lncRNA data for early longan SE suggested the lncRNAs were involved in regulation at each SE stage.

### Longan lncRNA target prediction and functional annotation

The potential target genes of the lncRNAs were predicted according to their regulatory methods, which were divided into *cis*- and *trans*-regulation. Of the 6005 lncRNAs, only 4682 lncRNAs were predicted to target protein-coding genes, including 5051 *cis*-regulated target genes (5712 pairs) and 1605 *trans*-regulated target genes (3618 pairs) (Additional file 3).

**Fig. 3:**
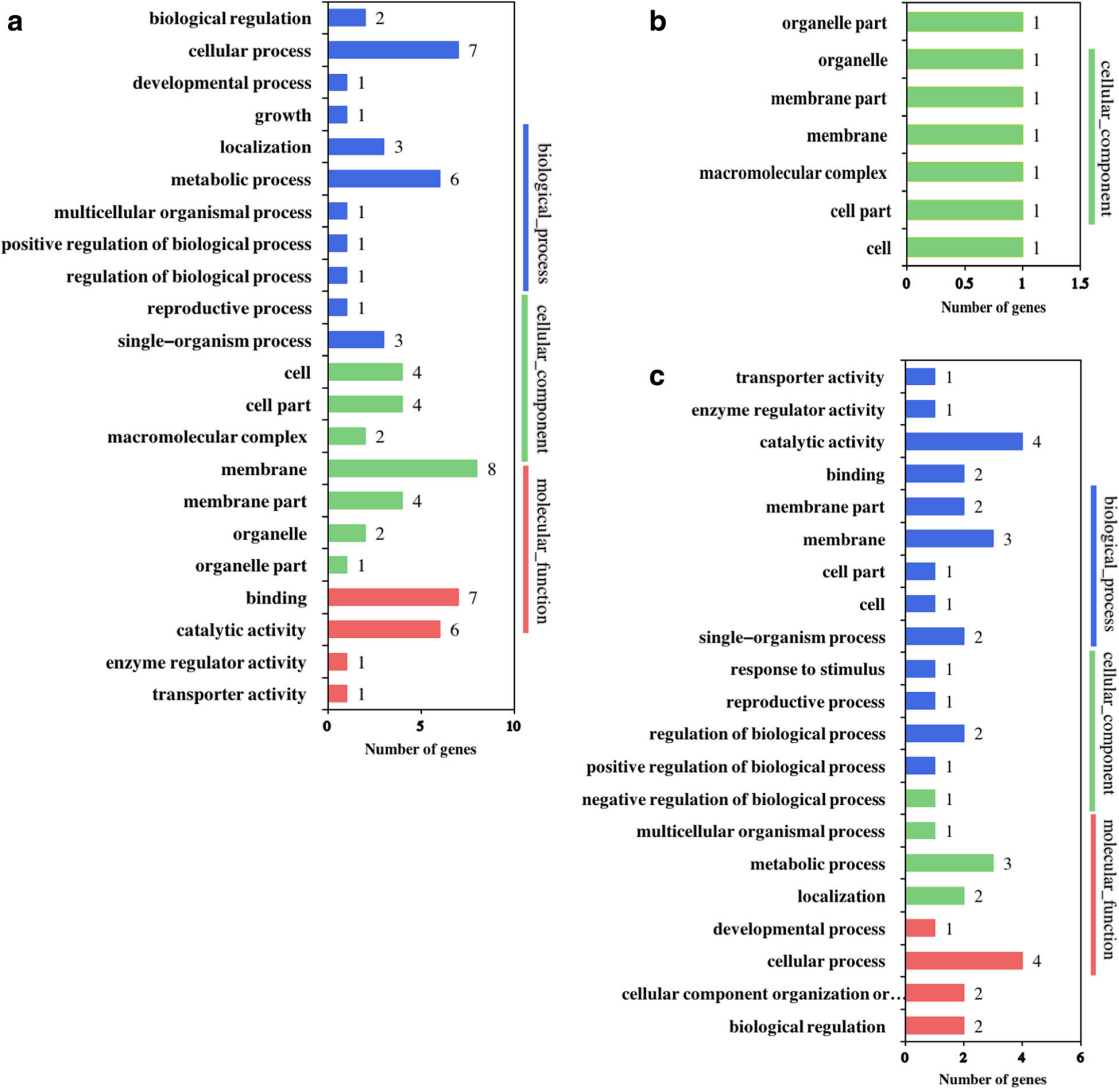
Gene ontology analysis of differentially expressed genes (DEGs) of potential lncRNA target genes under the molecular function, cellular component and biological processes categories. **a** EC vs. GE, (**b**) EC vs. ICpEC, and (**c**) ICpEC vs. GE

Among the potential target genes (mRNAs) of the lncRNAs, 852 genes were deferentially expressed in EC vs. GE (Additional file 4), with 558 up-regulated and 294 down-regulated. There were 538 differentially expressed genes in ICpEC vs. GE, among which 384 were up-regulated and 154 were down-regulated. The number of differentially expressed genes in EC vs. ICpEC was 99, among which 34 were up-regulated and 55 were down-regulated. Gene Ontology (GO) analysis was performed to investigate the differentially expressed target genes in the EC, ICpEC and GE stages (Fig. 3); an FDR ≤ 0.01 was considered to indicate significant enrichment. Only the cellular component genes were enriched in EC vs. ICpEC. In ICpEC vs. GE, a total of 31, 9, and 8 genes were assigned to the biological process, cellular component, and molecular function categories, respectively. In EC vs. GE, genes in the biological process, cellular component, and molecular function categories were also enriched. In the biological process category, 32 terms including protein phosphorylation, RNA metabolic process, and transport were significantly enriched, while in the cellular component category, the top three terms were membrane, protein complex, and nucleus. In the molecular function category, kinase activity, transferase activity/transferring phosphorus-containing terms and ATP binding were the most abundant terms.

We further categorized the 39,119 differentially expressed potential target genes (mRNAs) using Kyoto Encyclopedia of Genes and Genomes (KEGG) (Additional file 5). The results indicated that 51 potential target genes were enriched in 23 pathways in EC vs. ICpEC; the top five most enriched pathways were as follows: linoleic acid metabolism, steroid biosynthesis, aminoacyl-tRNA biosynthesis, ubiquitin mediated proteolysis, and base excision repair. In ICpEC vs. GE, 424 potential target genes were enriched in 77 pathways and the top five most enriched pathways included: plant-pathogen interactions, plant hormone signaling, glyoxylate and dicarboxylate metabolism, homologous recombination, and brassinosteroid biosynthesis. Additionally, 686 potential target genes were enriched in EC vs. GE; the EC to the GE stage is the general period of early development of longan SE. Among all 89 enriched KEGG pathways, 92 potential target genes of lncRNAs were related to “plant-pathogen interaction” and 30 target genes were related to “plant hormone signaling”. The top five most enriched pathways included: plant-pathogen interaction, plant hormone signal transduction, sulfur metabolism, homologous recombination and linoleic acid metabolism. Plant-pathogen interaction, plant hormone signal transduction, homologous recombination, and linoleic acid metabolism were significantly affected during the ICpEC and GE stages compared with the EC stage. Five genes related to auxin response factors that were targeted by lncRNAs were identified: an auxin (AUX) gene targeted by LTCONS-00006334; auxin response factor (ARF) genes targeted by LTCONS-00025525, LTCONS-00030223, and LTCONS-00055024; and an auxin-responsive protein IAA (AUX/IAA) gene targeted by LTCONS-00008111. These targets of differentially expressed lncRNAs, therefore, were considered to play fundamental roles in early longan SE.

### FPKM-qPCR comparative analysis of differentially expressed lncRNAs and their targets during longan early SE

To confirm the significant differences in the expression of lncRNAs and their predicted target genes in early longan SE, 20 lncRNAs with significant differences in expression were selected from among 641 up-regulated and 316 down-regulated lncRNAs in the RNA-Seq data. Additionally, 11 potential target genes (mRNAs) and five lncRNAs related to auxin response factors were verified by qPCR in the three stages of early longan SE. Twenty-four of the lncRNAs were detected in EC, ICpEC and GE (excluding LTCONS-00027337).

**Fig. 4:**
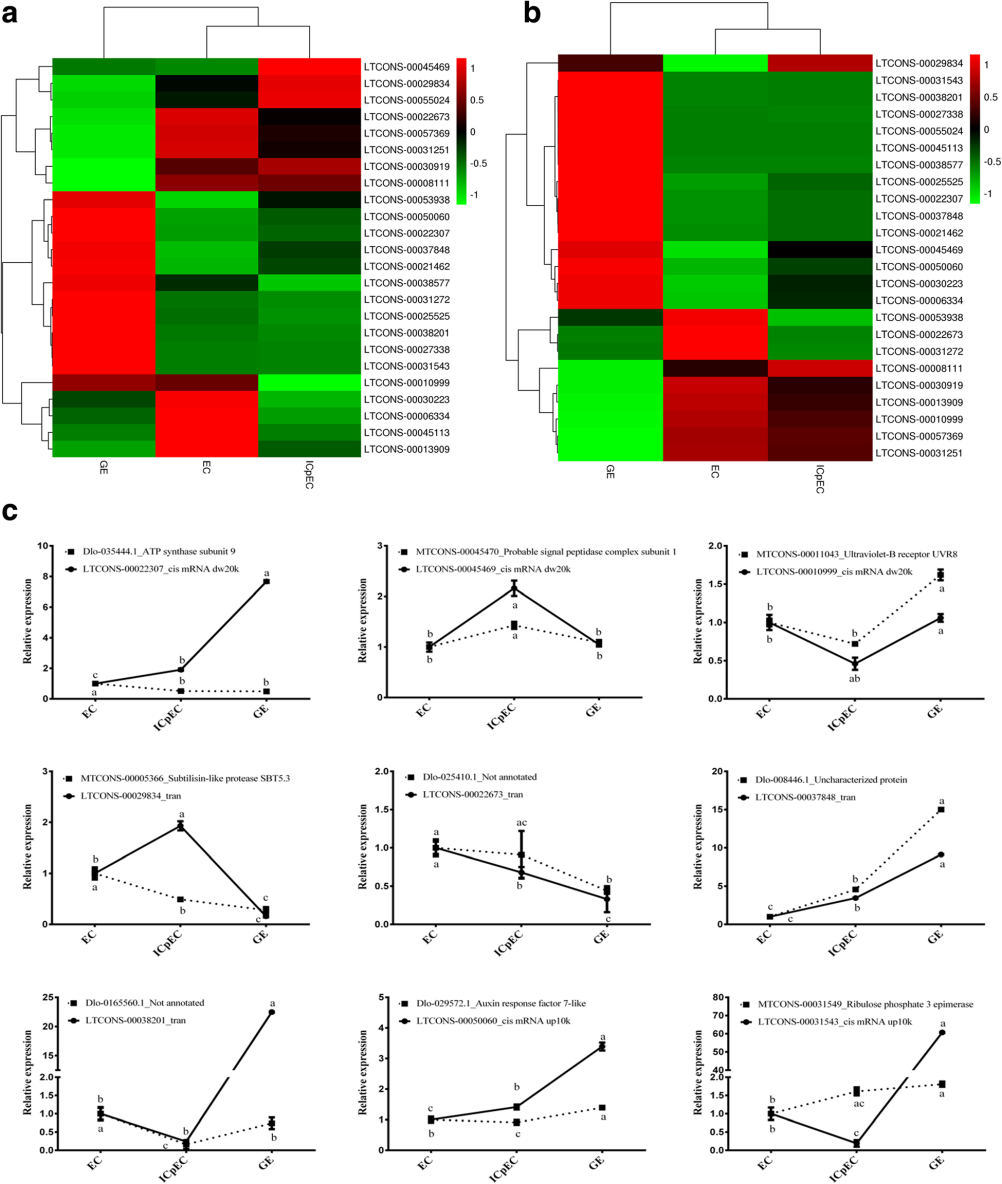
Analyses of differentially expressed lncRNAs and their targets in the RNA-Seq libraries and qPCR. Twenty-four differentially expressed lncRNAs were selected from the EC, ICpEC and GE samples. Heat maps were constructed for (**a**) relative expression levels in qPCR (**b**) and log2 (FPKM) values in the RNA-Seq library. The colors ranging from red to green indicate high to low correlation. **c** Expression patterns of nine lncRNAs corresponding to the heat map and their potential target genes. *DlRan3B* was used as a reference gene to normalize lncRNA and mRNA expression data

Cluster analysis of the expression patterns of the 24 lncRNAs in qPCR (Fig. 4a) and RNA-Seq (Fig. 4b), and qPCR of nine selected lncRNAs (Fig. 4c) was performed during early longan SE. The expression patterns of the 24 lncRNAs were divided into three categories according to the timing of their highest expression level. In category I, eight lncRNAs exhibited high levels in the EC stage, but low levels in the other two stages. LTCONS-00022673, LTCONS-00057369, LTCONS-00031251 and LTCONS-00008111 showed a decrease in expression from the ICpEC to GE stage, which indicated that their expression levels decreased gradually during longan SE, while the expression levels of LTCONS-00030223, LTCONS-00006334, LTCONS-00045113 and LTCONS-00013909 did not change much from the ICpEC to GE stage. In category II, four lncRNAs had the highest expression in the ICpEC stage. The expression levels of LTCONS-00030919 and LTCONS-00045469 in the EC and GE stages were basically unchanged. The expression levels of LTCONS-00029834 and LTCONS-00055024 in the EC stage were higher than in the GE stage. In category III, the expression of the 12 lncRNAs was highest in the GE stage; the expressions of LTCONS-00038577 and LTCONS-00010999 decreased gradually from the EC to ICpEC stage, while that of LTCONS-00031272, LTCONS-00025525, LTCONS -00038201, LTCONS-00027338 and LTCONS-00031543 did not change much at the EC and ICpEC stages. The expressions of LTCONS-00053938, LTCONS-00050060, LTCONS-00022307, LTCONS-00037848 and LTCONS-00021462 increased gradually from EC to ICpEC. These results indicated that different lncRNAs might be involved in the maintenance of different stages of longan SE, i.e. the eight lncRNAs of category I could be related to maintaining the pre-embryonic status of the EC, the four lncRNAs of category II could be related to maintaining the embryogenic status of the ICpEC, and the 12 lncRNAs of category III could be related to maintaining the embryonic status of the GE.

Comparing the expression trends of the lncRNAs in qPCR (Fig. 4a) with those in RNA-Seq (Fig. 4b) showed that the expression trends of 16 of the 24 lncRNAs were the same. The inconsistencies of the other eight lncRNAs were as follows: LTCONS-00053938 showed highest expression in the GE stage and the lowest expression in the EC stage according to qPCR, but its expression was lowest in the GE stage and highest in the EC stage in RNA-Seq. The expression level of LTCONS-00010999 decreased from the EC to ICpEC to GE stage in qPCR, but increased from the EC to ICpEC to GE stage in RNA-Seq. The expression of LTCONS-00031272 in the GE stage was higher in qPCR than in RNA-Seq. For LTCONS-00030223, LTCONS-00006334 and LTCONS-00045113, the expression in qPCR was highest in the EC stage and in RNA-Seq was highest in the GE stage. The expression of LTCONS-00055024 and LTCONS-00045469 was highest in the ICpEC stage in qPCR, but highest in the GE stage in RNA-Seq.

In the qPCR analysis of 11 lncRNAs and their potential target genes (mRNAs), except for LTCONS-00013909 and the target gene MTCONS-00013943 of LTCONS-00027337, all of the lncRNAs and their target genes were detected (Fig. 4c). According to the expression patterns of the lncRNAs and their target genes mRNAs, LTCONS-00022307, LTCONS-00045469 and LTCONS-00010999 were found to target *cis* mRNAs 20 kbp downstream (*cis* mRNA dw20k), while LTCONS-00029834, LTCONS-00022673, LTCONS-00037848 and LTCONS-00038201 were *trans*-acting, and LTCONS-00050060 and LTCONS-00031543 targeted *cis* mRNAs 10 kbp upstream (*cis* mRNAs up10k).

Among the three *cis* mRNA dw20k-type lncRNAs, only LTCONS-00022307 negatively regulated its potential target genes during early longan SE; the other two lncRNAs were positive regulators. Among the four *trans*-acting lncRNAs, LTCONS-00022673, LTCONS-00037848 and LTCONS-00038201 had a significant positive correlation with their target genes. Of the two *cis* mRNAs up10k lncRNAs, LTCONS-00031543 had a positive regulatory effect on its target gene. From the above results, we deduced that the regulation of target genes by the lncRNAs did not entirely depend on the positional relationship between the lncRNAs and their target genes.

### Functions of lncRNAs in the lncRNA-miRNA expression network in longan early SE

There were a large number of differentially expressed miRNAs and lncRNAs in longan SE, so to study the regulatory roles of lncRNAs in longan SE, we focused on three aspects, including lncRNAs acting as eTMs for miRNAs, lncRNAs acting as precursors to miRNAs and lncRNAs as miRNA target genes.

**Fig. 5:**
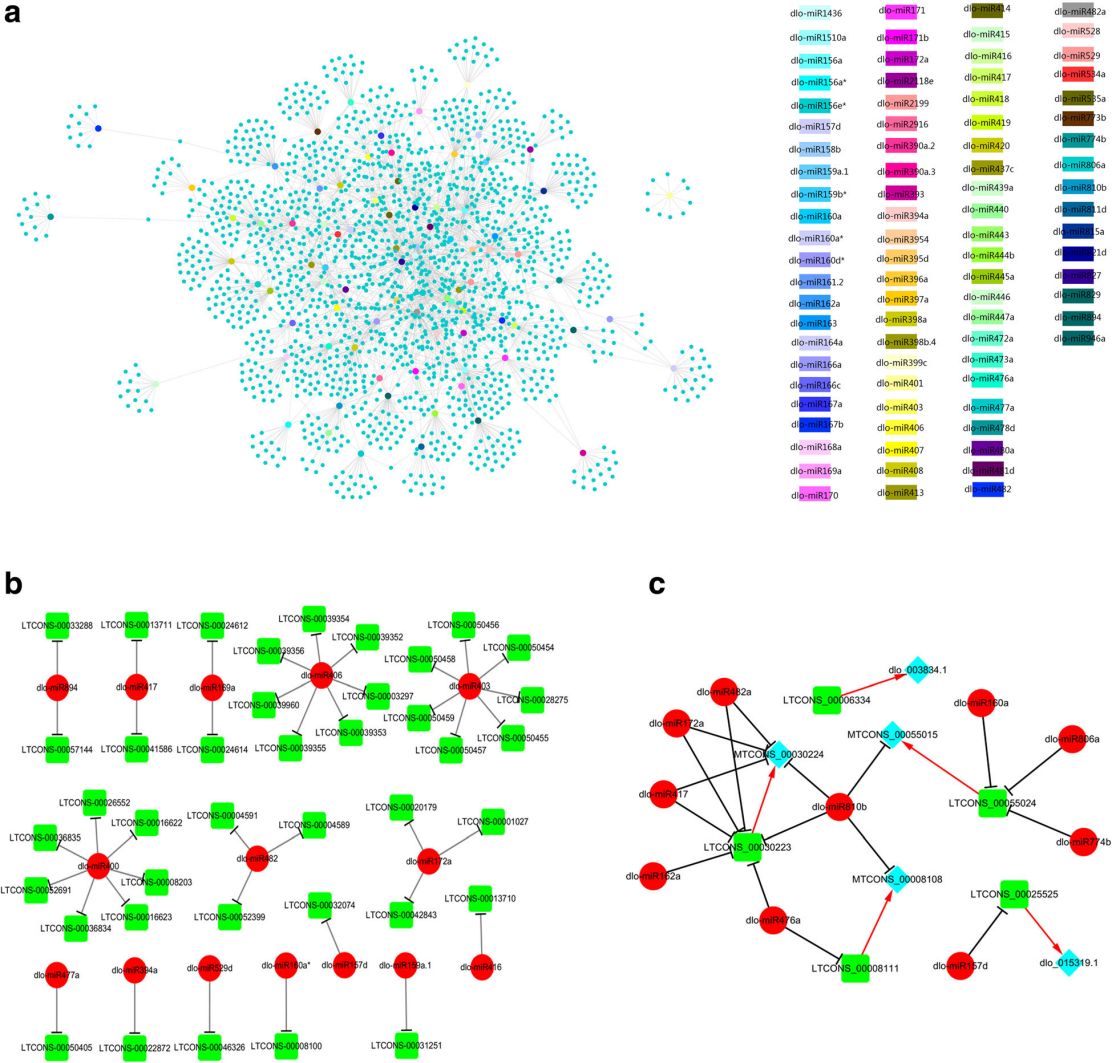
Prediction of lncRNA-miRNA-mRNA relationships in early longan SE. **a** In total, 1765 lncRNAs were predicted as targets of 85 miRNAs by psRNATarget (expectation ≤5). **b** Forty lncRNAs were predicted to function as eTMs for 15 miRNAs by TAPIR. **c** Five lncRNAs involved in auxin response factor mRNA and miRNA networks. The black arrows indicate relationships between lncRNAs and miRNAs and the red arrows indicate relationships between lncRNAs and mRNAs

As a new type of regulatory factor, eTMs play an important regulatory role in the process of plant cell differentiation and development [25, 35]. By binding a miRNA, an eTM blocks miRNA cleavage and thus isolates the miRNA from its target mRNAs. In the present work, 40 lncRNAs were predicted to function as eTMs for 15 miRNAs (Fig. 5b) with TAPIR. Based on their sequence conservation, these 15 miRNAs came from 15 different miRNA families, including miR403, miR406 and miR400. Because miRNAs have a significant regulatory effect on their target mRNAs, we speculated that the lncRNAs acting as eTMs for miRNAs during early longan SE could greatly affect the miRNA regulatory network.

Genome-wide studies have shown that a significant fraction of lncRNAs may function as precursors of small RNAs < 200 nucleotides (nt) in length. Of the 7643 predicted lncRNAs, seven were identified as potential miRNA precursors of three families (Table 1). LTCONS-00010286, LTCONS-00010359 and LTCONS-00010360 might serve as precursors of the miR162 family, LTCONS-00022889 was predicted to be a precursor of the miR319 family, and LTCONS-00025684, LTCONS-00025685 and LTCONS-00032074 might serve as precursors to the miR156 family. During target gene prediction and annotation for these seven lncRNAs, only LTCONS-00025685 and LTCONS-00032074 were found to have potential target mRNAs, which were annotated as *TFIID* and eukaryotic initiation Factor 4A-11.

**Table 1:** LncRNAs corresponding to miRNA precursors

lncRNAs ID	miRNA ID	Length of lncRNAs	identity	Evalue
LTCONS-00010286	rco-MIR162	3447	91	2.00E-28
	mes-MIR162	3447	92.39	8.00E-28
LTCONS-00010359	rco-MIR162	2564	91	2.00E-28
	mes-MIR162	2564	92.39	8.00E-28
LTCONS-00010360	rco-MIR162	583	91	2.00E-28
	mes-MIR162	583	92.39	8.00E-28
LTCONS-00022889	lus-MIR319a	2612	87.15	5.00E-40
	ppe-MIR319b	2612	84.55	2.00E-16
	mes-MIR319b	2612	91.62	4.00E-59
LTCONS-00025684	tcc-MIR156g	5776	99.09	4.00E-55
	gma-MIR156h	5776	92.05	2.00E-25
	gma-MIR156k	5776	91.3	5.00E-23
	vun-MIR156a	5776	90.43	3.00E-24
	mes-MIR156e	5776	94.44	8.00E-34
	ppe-MIR156e	5776	90.48	2.00E-18
LTCONS-00025685	tcc-MIR156g	6221	99.09	4.00E-55
	gma-MIR156h	6221	92.05	2.00E-25
	gma-MIR156k	6221	91.3	5.00E-23
	vun-MIR156a	6221	90.43	3.00E-24
	mes-MIR156e	6221	94.44	8.00E-34
	ppe-MIR156e	6221	90.48	2.00E-18
LTCONS-00032074	ptc-MIR156b	5681	88.46	3.00E-21
	mdm-MIR156b	5681	90.7	2.00E-23
	mdm-MIR156o	5681	95.56	9.00E-34
	mes-MIR156c	5681	87.78	6.00E-16

Some of the lncRNAs responding to early longan SE might be regulated as targets by miRNAs. The “psRNATarget” program (expectation ≤5) was used to predict the possible roles of miRNA targets among the 7643 lncRNAs (Additional file 6). In total, 1765 lncRNAs were predicted to be targets of 85 miRNAs in 74 families. One lncRNA could be targeted by multiple miRNAs. For example, LTCONS-00000242 was targeted by miRNAs belonging to six different families. Conversely, one miRNA could also target multiple lncRNAs, e.g. Dlo-miR1436 was predicted to target 182 different lncRNAs. Based on the above predictions, a miRNA targeting regulatory network for the lncRNAs was constructed (Fig. 5a), and it was speculated that a large number of lncRNAs were regulated as targets of miRNAs during early longan SE.

### Relationship between lncRNAs and potential target genes involved in auxin signal transduction

Studies have shown that auxin plays a regulatory role in plant embryogenesis and regulates embryo development via the regulation of auxin-responsive transcription [36, 37]. In KEGG enrichment analysis of the 39,119 differentially expressed potential target genes during the early development of longan SE, 30 differentially expressed genes (5.33% of genes annotated to the pathway) in EC vs. GE, 2 differentially expressed genes (2.67%) in EC vs. ICpEC, and 31 differentially expressed genes (8.33%) in ICpEC vs. GE were enriched in the phytohormone signaling pathway. Five target genes in the auxin signaling pathway were identified by lncRNA target gene prediction, including *ARF4* targeted by LTCONS-00025525, *IAA6* and *AUX22* targeted by LTCONS-00008111, *ABF* targeted by LTCONS-00030223 and LTCONS-00055024, and *AUX2* targeted by LTCONS-00006334 (Fig. 5c).

**Fig. 6:**
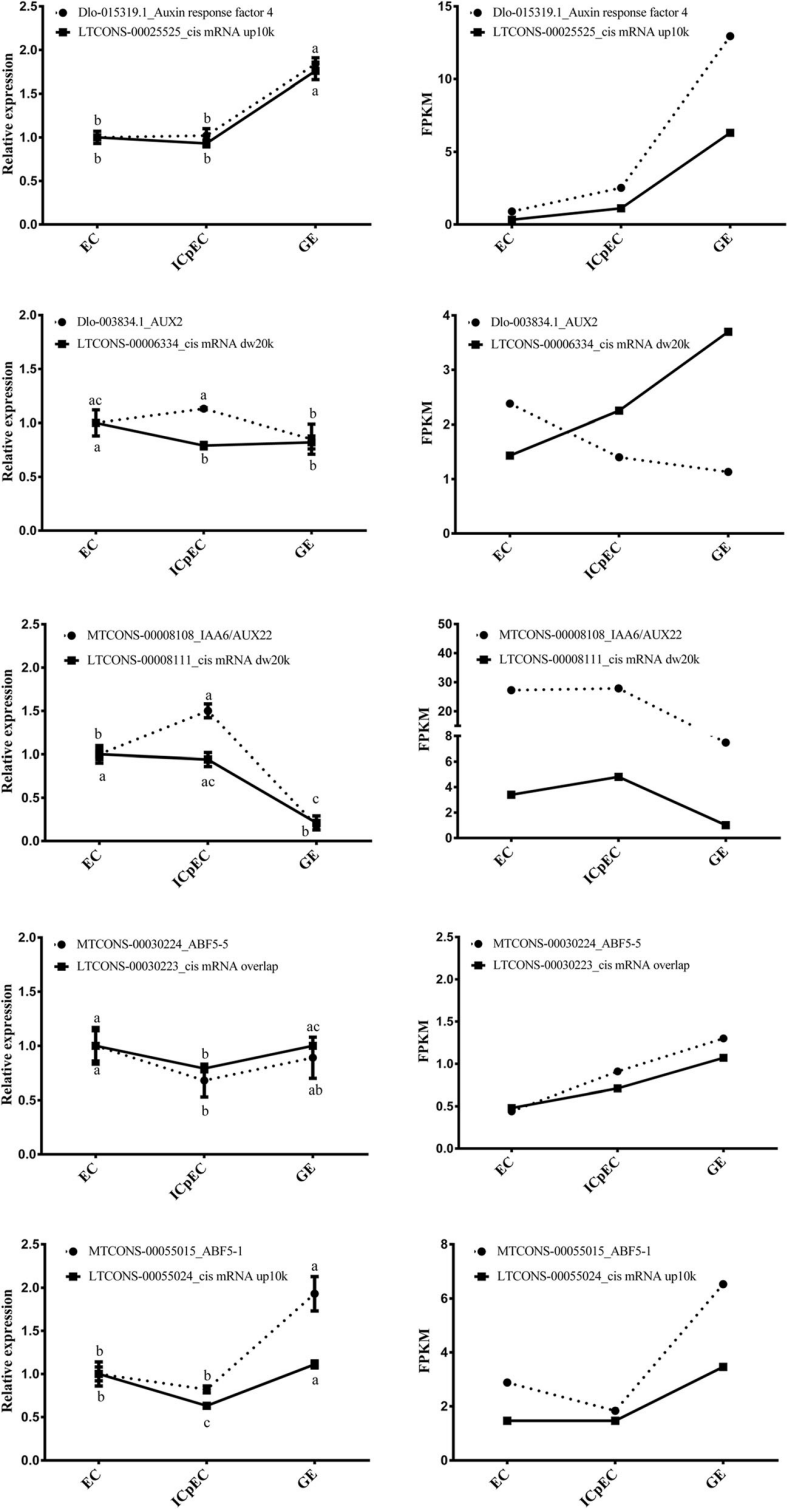
Expression of five lncRNAs that regulate auxin response factors in RNA-Seq and qPCR. Most of the five genes showed the same trend. *FSD* was used as a reference gene to normalize lncRNA and mRNA expression data

To profile the expression patterns of the lncRNAs associated with auxin response factors, five lncRNAs, their target genes (auxin response factors) and the corresponding miRNAs were analyzed by qPCR in EC, ICpEC and GE (Fig. 6). Although there were some differences in expression levels, the qPCR and RNA-Seq data showed the same changes trends. The qPCR results showed that LTCONS-00025225 regulated *ARF4* and LTCONS-00055024 regulated *ABF5–1*, both of which were *cis* mRNA up10k types and were highly expressed in GE. LTCONS-00006334 regulated *AUX*2 and LTCONS-00008111 regulated *IAA6/AUX22*, and these target genes were *ci*s mRNA dw20k types. LTCONS-00030223 regulated *ABF5–5*, which was an overlapping *cis* mRNA, and both were highly expressed in EC. While there was a negative regulatory relationship between LTCONS-00006334 and its target gene, the other four lncRNAs positively regulated their target genes. Thus, lncRNAs with different modes of action were involved in the auxin signaling pathway, with different potential target genes and corresponding differential expression patterns in different stages of longan SE, which indicated that lncRNAs were involved in the auxin signaling pathway through complex regulatory mechanisms.

In summary, the lncRNA-miRNA-mRNA regulatory network in the auxin signaling pathway was constructed from lncRNAs, miRNAs and mRNAs, and verified by qPCR of five lncRNAs and related miRNAs (Additional file 7). The results showed that only related miRNAs were differentially expressed during the early development of longan SE, including Dlo-miR157d, which was related to LTCONS-00025525 targeting *ARF4*, Dlo-miR482a and Dlo-miR482a, which were related to LTCONS-00030223 targeting *ABF5–5*, and miR172a, Dlo-miR417, Dlo-miR162a^*^, and Dlo-miR806a, which were associated with LTCONS-00055024 targeting *ABF5–1*. The remaining miRNAs showed no significant difference during early longan SE.

### Overexpression and inhibition of miR172a, miR159a.1 and miR398a in longan embryogenic callus

**Fig. 7:**
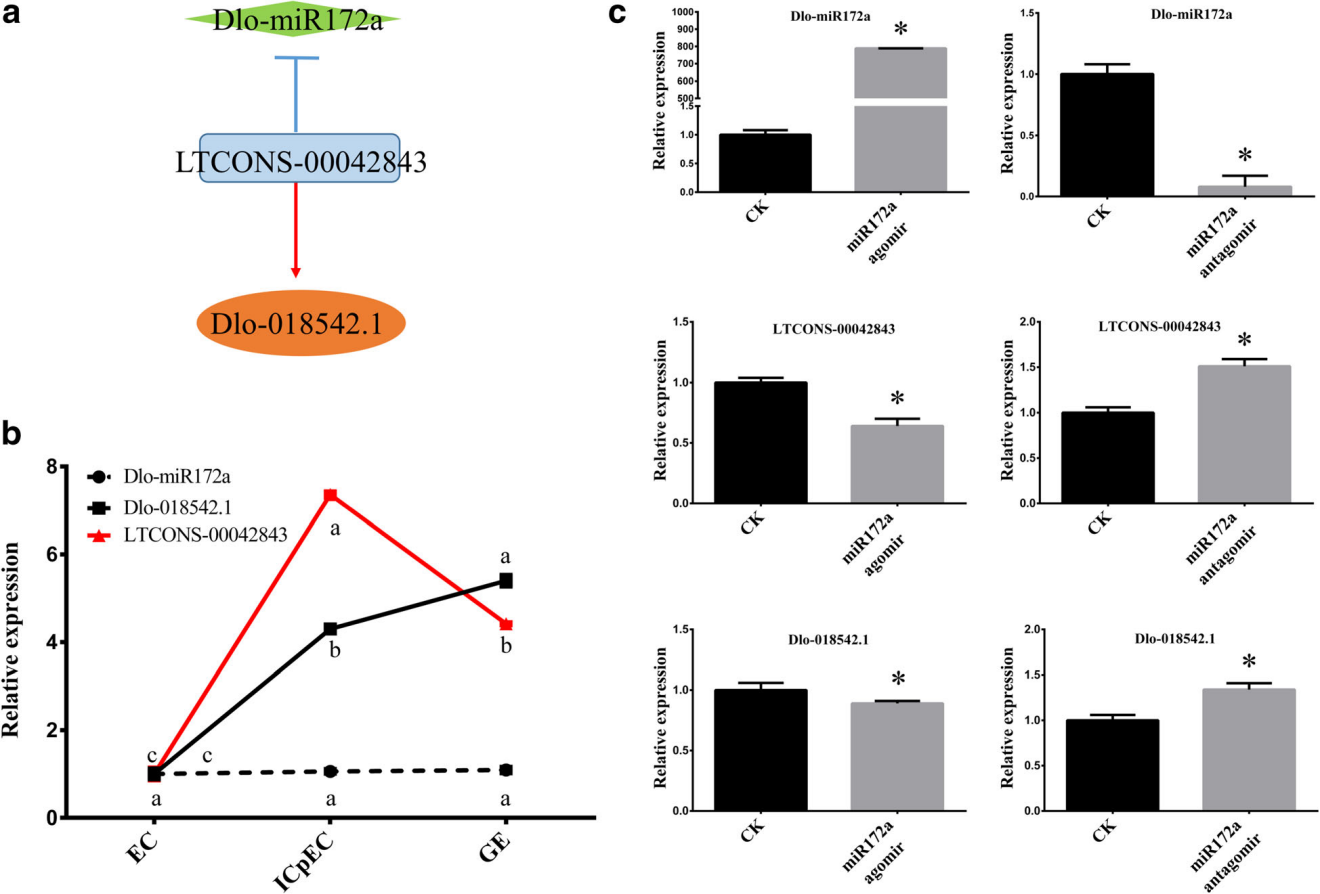
qPCR validation of the Dlo-miR172a lncRNA-miRNA-mRNA network. Dlo-miR164a was used as a reference gene to normalize miRNA expression data; *DlRan3A* was used to normalize lncRNAs and mRNAs. **a** LTCONS-00042843 targeted Dlo-018542.1 and acted on miR172a as an eTM. The blue arrows indicate a relationship between lncRNAs and miRNAs as predicted eTMs and the red arrows indicate a relationship between lncRNAs and mRNAs as predicted targets. **b** qPCR expression trends of Dlo-miR172a, LTCONS-00042843 and Dlo-01842.1 during early longan SE. **c** Dlo-miR172a overexpression inhibited lncRNA-miRNA-mRNA expression in the sample material

After predicting the relationships between lncRNAs, miRNAs and mRNAs, three networks of miRNAs closely related to SE (miR172a, miR159a.1 and miR398a) were selected for qPCR verification in longan. In the miR172a network, LTCONS-00042843 targeted Dlo-018542.1 and acted on miR172a as an eTM (Fig. 7a). The expression of Dlo-miR172a tended to be stable during the early stages of longan SE with no significant changes. However, the expressions of LTCONS-00042843 and Dlo-018542.1 was significantly different in ICpEC and GE compared with the EC stage. LTCONS-00042843 had the highest expression level at the ICpEC stage, while Dlo-018542.1 had the highest expression level at the GE stage (Fig. 7b). Expression comparisons between LTCONS-00042843 and Dlo-018542.1 under overexpression and inhibition of miR172a in longan EC were conducted, and the results showed that the expression of LTCONS-00042843 and Dlo-018542.1 decreased when Dlo-miR172a was overexpressed but increased when Dlo-miR172a expression was inhibited compared with the control check (CK) (Fig. 7c), which was consistent with the predicted relationships among lncRNAs, mRNAs and miRNAs in longan SE. and indicated that inter-regulation among them might exist.

In the miR159a.1 network (Additional file 8), LTCONS-00046326 was predicted to be a Dlo-miR159a.1 target gene and to act as an eTM for Dlo-miR529d, and both Dlo-017525.1 and Dlo-028266.1 were predicted to be target genes of LTCONS-00046326. Surprisingly, both overexpression and inhibition of Dlo-miR159a.1 promoted the expression of the target gene LTCONS-00046326, implying the existence of a complicated regulatory network of lncRNA, miRNA and mRNA.

In the miR398a network (Additional file 9), Dlo-miR398a acted on the target genes LTCONS-00032074 and LTCONS-00039356, which in turn acted as eTMs for Dlo-miR157d and Dlo-miR406, respectively. Using primers targeting different sites, it was found that the expression level of LTCONS-00039356, a target gene of Dlo-miR398a, was not significantly different from that of CK under overexpression and inhibition of Dlo-miR398a in longan EC; however, LTCONS-00039356, as an eTM of Dlo-miR157d and Dlo-miR406, showed significantly different expression compared with CK. There was a significant change in response tendencies with overexpression and inhibition of Dlo-miR398a in longan EC, but Dlo-miR157d and Dlo-miR406 showed no significant response trends in the same material. The above results shows that lncRNAs bound to miRNAs at different binding sites and had different expression in the same material.

## Discussion

### Longan may require a large amount of lncRNA to initiate GE formation

Many researchers have focused on lncRNAs in recent years, and remarkable progress has been made, especially in human and animals [38]. Conversely, studies on lncRNAs in plants are still in their infancy, and most have focused on crops such as model plants [11, 39] and rice [40]. To date, there have been no reports on the involvement of lncRNAs in plant SE; thus, the present work is the first genome-wide identification and characterization of lncRNAs in plant SE. Here, 6005 novel lncRNAs were identified as associated with early longan SE, i.e. the EC, ICpEC and GE stages. Compared with lncRNA identification in other horticultural plants, e.g. cucumber [41] (fruit and root; 3274 lncRNAs), sunflower [42] (early flower bud; 25,327 lncRNAs), *Brassica napus* [43] (leaf; 9880 lncRNAs), the number of lncRNAs obtained from early longan SE was moderate, the poor conservation of lncRNAs among different species and the different tissues might be an important reason for the significant differences in the number of lncRNAs identified during early SE. In the three stages of early longan SE, 375 lncRNAs were specifically expressed in the GE stage, which was much more than in the EC stage (159) and the ICpEC stage (153). Among the three stages of the longan SE, the number of differentially expressed lncRNAs was highest in the EC vs. GE, and the number of differentially expressed lncRNAs in ICpEC vs. GE was 4.55 times that in EC vs. ICpEC. Thus, in longan SE, the establishment of embryo morphology and structure requires more lncRNA involvement, and achieving embryonic status (GE) requires significantly different molecular mechanisms to omnipotence maintenance (EC) and embryogenesis initiation (ICpEC).

### lncRNAs are mainly involved in plant-pathogen interaction and phytohormone signaling pathways during longan early SE

In total, 74.39% of the lncRNAs involved in the early stages of longan SE had only one or two exons, which might account for their low expression levels. The conservation of lncRNAs among species is poor, which also hinders research on lncRNAs. It has been reported that lncRNAs play a regulatory roles by acting on protein-coding genes [44, 45]. Therefore, differential enrichment analysis of the target genes (protein-coding genes) of lncRNAs during early longan SE provided valuable information. In this study, the plant-pathogen interaction, plant hormone signaling, sulfur metabolism and various kinds of amino acid metabolism pathways were revealed to be closely related to early longan SE. Of the top five most enriched pathways, three were commonly enriched in EC vs. GE and ICpEC vs. GE, and the number of differentially expressed lncRNAs in ICpEC vs. GE was 4.55 times that in EC vs. ICpEC. These results indicate that similar molecular mechanisms are active between the pre-embryonic EC and the embryogenic ICpEC, and the main change leading to GE formation occurs in the ICpEC to GE stages. Thus, it can be seen that the molecular mechanisms of embryo formation can change dramatically.

Previous studies reported that the plant-pathogen interaction pathway was enriched in lncRNAs [46], miRNAs [47, 48] and gene transcripts [23], which suggested the importance of plant-pathogen interactions in the embryonic developmental network in longan and other plants. Plant hormones play important roles in plant growth and SE in plants [49]. In a study of SE of *Hevea* seeds, it was found that an excess of auxin and cytokinin inhibited the embryogenic ability of callus [50]. Zhu et al. studied the effects of plant hormones on SE and plant regeneration in cassava (*Manihot esculenta* Crantz) [51]. The phytohormone signal transduction pathway includes auxin, kinetin, gibberellin and abscisic acid, among which auxin is an important factor in plant SE, e.g. it regulates embryonic development by regulating *ARF*-mediated transcription [36, 37], and *Aux/IAAs* also participate in the regulation of auxin by inhibiting *ARF*-mediated transcription [52]. Our laboratory has studied related genes including *TIF1* [53], *DlARF8* [17], *ARF3/4* [18] and *ARF10*, *− 16*, and *− 17* [19] that regulate the auxin response. In the KEGG enrichment results, it seemed that longan had begun to establish disease resistance mechanisms and other metabolic components based on hormones in the GE stage. This change not only involved a large number of mRNAs, but also a large number of lncRNAs that participate in the molecular regulation and morphogenesis of the GE stage by regulating target genes (mRNA).

lncRNAs regulate the expression of target genes in *cis* or *trans* through a variety of mechanisms. Previous studies have shown that *cis* regulation by lncRNAs includes enhancer activity [54] and insulator functions [55]. A common method of *trans*-regulation is the upregulation of target gene expression by forming dimers with neighboring mRNAs that increase mRNA stability [56, 57]. In this study, the locations of 14 pairs of lncRNAs and their target mRNAs were verified by qPCR analysis. Ten pairs of *cis*-regulated mRNAs and lncRNAs were identified, including seven pairs that had positively regulated and three pairs that had negatively regulated genes. Four pairs of *trans*-regulated mRNAs and lncRNAs were found in longan SE, including three pairs with positive regulatory relationships and one pair with a negative regulatory relationship. The target genes of the five lncRNAs associated with auxin response factors were *cis*-regulated, which implied that the lncRNAs associated with auxin response factors mainly function as *cis*-acting regulators during early longan SE.

### lncRNAs acting as eTMs for miRNAs may be an important regulatory mechanism during longan early SE

lncRNAs were observed to function as eTMs for miRNAs, revealing a new mechanism for the regulating of miRNAs by lncRNAs in plants. lncRNAs bind to miRNAs as endogenous trapping targets of miRNAs and reduce the miRNAs’ function to inhibit target expression. In Arabidopsis, the lncRNAs *IPS1* bound to ath-miR399 and a three-nucleotide bulge was formed between the 10th and 11th positions of the 5 ‘end to prevent the cleavage of *IPS1* by ath-miR399 [11]. In addition, miR160a* largely interacted with its predicted eTMs during the GE and EC stages [19], and the interaction between miR172 and eTMs could delay flowering and eliminate top-of-the-range benefits [58, 59]. MiRNAs and their predicted eTMs have also been reported during longan SE: miR167 cleaved the *DlARF8* mRNA during longan SE [17], and an eTM down-regulated miR167, and the regulation of miR160 and its predicted eTM were involved in hormone signaling in longan [19].

In our study, 40 lncRNAs were predicted to function as eTMs for 15 miRNAs and four lncRNAs were validated as eTMs of four miRNAs (Dlo-miR172a, Dlo-miR529d, Dlo-miR157d, and Dlo-miR406). LTCONS-00042843, LTCONS-00046326, LTCONS-00032074, and LTCONS-00039356 were respectively identified as eTMs for Dlo-miR172a, Dlo-miR529d, Dlo-miR157d, and Dlo-miR406. Additionally, the qPCR results showed that LTCONS-00042843 and LTCONS-00046326 inhibited the expression of Dlo-miR172a and Dlo-miR529d, respectively. Our study is the first to discover that lncRNAs act as eTMs of miR172a and miR529d in the EC stage in longan.

### Multiple regulatory mechanisms may exist among lncRNA, miRNA and mRNA during early SE in longan

In addition to the above lncRNAs acting as eTMs for miRNAs, lncRNAs may also interact with miRNAs as miRNAs precursors and act as miRNAs target genes. During the early stages of longan SE, only seven lncRNAs were predicted to be precursors of miRNAs (miR156 family, miR319 family and miR162 family). In longan SE, members of the Dlo-miR156 family were associated with different developmental stages of early embryo cultures [16], and Dlo-miR162 regulated specific stages of SE through multiple miRNAs [60]. miR319b.1 was significantly accumulated at the cotyledon stage in lily SE [61]. In addition, miR156 regulated rice seed and pollen development [62], miR319 affected leaf development in *Arabidopsis* [63] and participated in rice stress responses [64], and miR162 might be involved in gene expression regulation and cotyledon formation during germination in Asteraceae. Functional annotation of target genes predicted for the lncRNAs suggested that Dlo-015497.1 and Dlo-019109.1 might be involved in transcriptional initiation as transcription initiation factors. Based on the functions of miRNAs and mRNAs in longan and other plants, we speculate that some lncRNAs are involved in regulation of gene expression by acting as miRNA precursors during early longan SE.

In the present work, based on miRNA target prediction, 1765 lncRNAs were predicted to be targets of 85 miRNAs of 74 families. Thus, the majority of lncRNAs regulation might be achieved via miRNAs during early longan SE, and lncRNAs might indirectly regulate the expression of mRNAs by regulating the number of miRNAs. Five lncRNAs related to the auxin response factors selected for miRNA target prediction, and seven miRNAs (lncRNAs as miRNA target genes) were predicted to be involved in the action of lncRNAs. Notably, both lncRNAs and t protein-coding genes could act on the same miRNA at the same time. For example, LTCONS-00030223 and MTCONS-00030224 are both target genes for Dlo-miR810b, Dlo-miR482a, Dlo-miR417 and Dlo-miR172a, and MTCONS-00030224 was predicted to be a target gene for LTCONS-00030223. Additionally, LTCONS-00055024 was a target gene for three miRNAs (Dlo-miR160a, Dlo-miR1774b and Dlo-miR806a). Studies have shown that dlo-miR160a targets *DlARF10*, *− 16* and *− 17* [19]. The target gene MTCONS-00055015 (*ABF-5*) of LTCONS-00055024 also regulates hormone signal transduction. Therefore, we speculate that LTCONS-00055024, MTCONS-00055015 and Dlo-miR160a might exist in a regulatory network.

To verify the relationships among lncRNAs, miRNAs and mRNAs during the early development of longan SE, qPCR was performed on Dlo-miR172a, Dlo-miR159a.1 and Dlo-miR398a. Through qPCR analysis of the EC samples with Dlo-miR172a, Dlo-miR159a.1 and Dlo-miR398a overexpression and inhibition, we found regulatory interactions of Dlo-miR172a and the corresponding lncRNA and mRNA during early SE in longan. However, the regulating relationshipS among Dlo-miR398a, Dlo-miR159a.1 and the corresponding mRNAs and lncRNAs were complicated and needed to be further explored.

## Conclusions

In this study, 6005 expressed lncRNAs were identified during the early stage of SE in longan. The number of lncRNAs specifically expressed in the GE stage was much greater than that in the EC stage and the ICpEC stage. We speculate that the GE stage requires more lncRNAs to regulate. The most highly enriched KEGG pathways were plant-pathogen interactions and phytohormone signaling. qPCR verification of five ARF-related lncRNAs and their target genes (mRNA) suggested that lncRNAs tend to positively regulate target genes in the early stages of longan SE. In lncRNA-miRNA-mRNA relationship predictions, some lncRNAs were predicted to function as eTMs for miRNAs and some were identified as potential miRNA precursors. In addition, we verified the lncRNA-miRNA-mRNA regulatory relationships by transient expression, and speculate that they comprise a regulatory network. Our results contribute to the understanding of lncRNA regulation in plant SE.

## Methods

### Plant materials

Synchronized embryogenic cultures at different developmental stages, consisting of friable-embryogenic callus (EC), incomplete compact pro-embryogenic cultures (ICpEC) and globular embryos (GE) were obtained following previously published methods for longan [15, 65]. The plant materials were stored at − 80 °C for later use.

### lncRNAs library construction and Illumina HiSeq sequencing

RNA samples were extracted from the three abovementioned cultures using Trizol reagent (Invitrogen, Carlsbad, CA, USA). The RNA was detected with an Agilent 2100 detection kit and the NanoDrop assay. The integrity of RNA was detected by 1.0% non-denaturing agarose gel electrophoresis. After the quality and concentration were checked, cDNA strands were synthesized with random primers and reverse transcriptase from the TruSeq® Stranded kit, and double-stranded cDNA was synthesized and amplified by using DNA polymerase I and RNaseH to obtain cDNA libraries. Raw reads were obtained using the Illumina HiSeq sequencing platform, and low-quality reads (quality Q ≤ 10 bases accounted for > 50% of the entire read), adapter contamination, and reads with a high content of N (containing an N ratio > 10%) were removed to get clean reads.

The clean reads were aligned to the longan reference genome [24] using the alignment software HISAT [66] for transcript assembly, and transcripts with lower expression (FPKM ≤0.5) and lengths below 200 bp were filtered out. The transcripts were identified as mRNAs or lncRNAs by using the software programs CPC [67], txCdsPredict and CNCI [68], and the Pfam database [69]. Inter-sample differential analysis of the obtained lncRNAs was carried out using the differential analysis method PossionDis [70] (fold change ≥2.00 and FDR ≤ 0.001), and by comparing the expression of the samples, genes with insignificant changes in expression were excluded and genes with significant differences were retained. The lncRNAs families were analyzed with NFERNAL [71], which annotates lncRNAs to families by aligning them to the Rfam database. The expression of the three samples was analyzed as a time series according to previous studies [72, 73].

### lncRNAs targets prediction and annotation

The potential target genes of the lncRNAs were predicted according to their regulatory methods, which were divided into cis- and trans-acting. The basic principle for the prediction of cis target genes is that the functions of lncRNAs are related to the protein coding genes near their coordinates [74]. Therefore, the neighboring mRNAs of lncRNAs were screened as target genes. *Trans* regulation is not dependent on positional relationships, and is predicted by calculating the binding energies [75]. To better understand the functions of the lncRNAs and their corresponding target genes, NT, NR, KOG, KEGG, and SwissProt annotations were assigned to the assembled novel and known mRNAs using Blast [76] or Diamond [77], Gene ontology (GO) annotations were assigned using Blast2GO [78] and NR, and InterPro annotations were assigned using InterProScan5 [79]. An FDR ≤ 0.01 was used to indicate significant enrichment.

### Prediction of the relationships between lncRNAs, miRNAs and mRNAs

Some lncRNAs can form miRNA precursors through intracellular shearing [80]. To identify lncRNAs as miRNA precursors during the early development of longan SE, the lncRNAs were aligned to miRBase [81] using Blast [77] (http://blast.ncbi.nlm.nih.gov/Blast.cgi). When looking for potential miRNA precursors, lncRNAs with miRNA precursor alignments greater than 90% were selected and considered precursor of lncRNAs. To predict lncRNAs as the target genes of miRNAs, the longan lncRNAs and miRNAs data were submitted to “psRNATarget” (http://plantgrn.noble.org/psRNATarget/) [82] with expectation ≤5. lncRNAs were predicted as eTMs for miRNAs and target trapping was identified by TAPIR (http://bioinformatics.psb.ugent.be/webtools/tapir/) [83], The Cytoscape_v3.5.1 software was used for network mapping of lncRNAs and related miRNAs and mRNAs.

### Real-time quantitative PCR

The 20 lncRNAs with the most significant differences were screened in four length intervals (0–500 bp, 500–1000 bp, 1000–1500 bp and 1500–2000 bp) of significant lncRNAs from the EC to GE stage, and specific primers were designed according to the obtained gene sequences. cDNA synthesis of cDNA was performed according to SMART™ RACE cDNA Amplification Kit and TransScript miRNA First-Strand cDNA Synthesis SuperMix Instruction Manual. Ten-fold dilutions of cDNAs from longan somatic embryos at three stages were used as templates for qRT-PCR amplification on a Roche LightCycler 480 instrument (Roche Applied Science, Switzerland). The gene qRT-PCR was carried out using a SYBR premix Ex Taq™ kit (TaKaRa) with a 20 μL reaction system containing, 10 μL SYBR, 1 μL cDNA template, 0.8 μL each of forward and reverse primers and 7.4 μL of ddH_2_O. The procedure was as follows: 95 °C predenaturation 30 s, 95 °C denaturation 10 s, 60 °C annealing 30 s, 72 °C extension 15 s, 40 cycles. miRNA qPCR using TransStart Tip Green qPCR SuperMix, the reaction system was 20 μL, the application of Tip 10 μL, cDNA template 1 μL, specific and universal primers 0.8 μL, ddH2O 7.4 μL, procedures: 95 °C predenaturation for 30 s and 40 cycles of 95 °C denaturation for 10 s, 60 °C annealing for 30 s, and 72 °C extension for 15 s. miRNA qPCR was performed using TransStart Tip Green qPCR SuperMix in a 20 μL reaction system containing 10 μL Tip Green SuperMix, 1 μL cDNA template, 0.8 μL specific and universal primers, and 7.4 μL ddH2O. The procedure was: 95 °C denaturation for 30 s and 40 cycles of 95 °C denaturation for 10 s, 60 °C annealing for 30 s, and 72 °C extension for 10 s. The 2^−ΔΔCt^ method was used to calculate expression values. The primers used for qPCR are listed in Additional file 9.

Heat maps of lncRNAs were produced using the online website Omicshare (http://www.omicshare.com). The heat maps were generated by Z-core processing of qPCR and RNA-Seq expression, which was the expression of each gene minus the mean of the gene in all samples and divided by the standard deviation.

### Transfection of miRNA inhibitors or miRNA mimics

Overexpression (agomir) and expression-inhibiting (antagomir) vectors of Dlo-miR172a, Dlo-miR159a.1 and Dlo-miR398a were synthesized by GenePharma. The Lipofectamine™ 2000 Transfection Reagent was used as a transfection reagent to transfer overexpression and expression-inhibiting carriers into longan calli [84]. The processing system was: 445 μL MS, 30 μL transfection agent and 25 μL carriers (overexpression or inhibition of expression). Dlo-miR172a, Dlo-miR159a.1 and Dlo-miR398a overexpression and inhibition of expression materials were obtained after 24 h dark culture.

## Additional files

Additional file 1: **Table S1:** Data filtering statistics. (XLS 65 kb)

Additional file 2: **Table S2.** lncRNA family analysis. (XLS 264 kb)

Additional file 3: **Table S3.** The potential target genes of lncRNAs. (XLS 1610 kb)

Additional file 4: **Table S4.** Significantly differentially expressed target genes. (XLS 233 kb)

Additional file 5: KEGG annotation analysis of potential target genes of lncRNAs. Functional categorization of potential target genes of lncRNAs based on the biological process category of the Kyoto Encyclopedia of Genes and Genomes (KEGG). (PDF 204 kb)

Additional file 6: **Table S5.** lncRNAs predicted as target genes of miRNAs. (XLS 1057 kb)

Additional file 7: qPCR validation of five miRNAs involved in “auxin response” regulation of lncRNAs. Dlo-miR172a was used as a reference gene to normalize miRNA expression data; *FSD* was used to normalize lncRNAs and mRNAs. (PDF 406 kb)

Additional file 8: qPCR validation of the lncRNA-miRNA-mRNA relationships of the miR159a.1 and miR398a networks. (a) miR159a.1 network qPCR validation. Dlo-miR396a was used as a reference gene to normalize miRNA expression data; *DlRan3A* was used to normalize lncRNAs and mRNAs. (b) miR398a network qPCR validation. Dlo-miR164a was used as a reference gene to normalize miRNA expression data; *EF-1α* was used to normalize lncRNAs and mRNAs. (PDF 1266 kb)

Additional file 9: **Table S6.** Primers used for real-time quantitative PCR. (XLS 64 kb)

## Abbreviations

ARF: Auxin response factor
cis mRNA dw20k: Cis mRNAs 20 kbp downstream
cis mRNAs up10k: Cis mRNAs 10 kbp upstream
CK: Control check
DL-ABP1: Longan auxin binding protein 1
DlLAC7: Longan laccase 7
DlPPO1: Longan polyphenol oxidase 1
DlTIR1–1: Longan transport inhibitor response 1–1
DlTIR1–2: Longan transport inhibitor response 1–2
DlWUS: Longan WUSCHEL
EC: Friable embryogenic callus
eTM: Endogenous target mimics
FPKM: Expected number of fragments per kilobase of transcript sequence per millions base pairs sequenced
GE: Globular embryo
GO: Gene ontology
ICpEC: Incomplete compact pro-embryogenic culture
KEGG: Kyoto Encyclopedia of Genes and Genomes
lncRNAs: Long non-coding RNAs
longan: *Dimocarpus longan* Lour
miRNA: MicroRNA
ORFs: Open reading frames
pol II: Polymerase II
pre-miRNA: Precursor miRNA
qPCR: Quantitative real-time PCR
qPCR: Real-time quantitative PCR

## References

[1] Wierzbicki AT (2012). The role of long non-coding RNA in transcriptional gene silencing. Curr Opin Plant Biol.

[2] Pruneski JA, Hainer SJ, Petrov KO, Martens JA (2011). The Paf1 complex represses SER3 transcription in Saccharomyces cerevisiae by facilitating intergenic transcription-dependent nucleosome occupancy of the SER3 promoter. Eukaryot Cell.

[3] Tripathi V, Ellis JD, Shen Z, Song DY, Pan Q, Watt AT, Freier SM, Bennett CF, Sharma A, Bubulya PA (2010). The nuclear-retained noncoding RNA MALAT1 regulates alternative splicing by modulating SR splicing factor phosphorylation. Mol Cell.

[4] Michael H, Nicholas D (2013). The intertwining of transposable elements and non-coding RNAs. Int J Mol Sci.

[5] Magistri M, Faghihi MA, Rd SLG, Wahlestedt C (2012). Regulation of chromatin structure by long noncoding RNAs: focus on natural antisense transcripts. Trends Genet.

[6] Johnsson P, Lipovich L, Grandér D, Morris KV (2014). Evolutionary conservation of long non-coding RNAs; sequence, structure, function. Biochim Biophys Acta.

[7] Cai X, Cullen BR (2007). The imprinted H19 noncoding RNA is a primary microRNA precursor. RNA.

[8] Wu HJ, Wang ZM, Wang M, Wang XJ (2013). Wide-spread long non-coding RNAs (lncRNAs) as endogenous target mimics (eTMs) for microRNAs in plants. Plant Physiol.

[9] Mohamed JS, Gaughwin PM, Bing L, Robson P, Lipovich L (2010). Conserved long noncoding RNAs transcriptionally regulated by *Oct4* and Nanog modulate pluripotency in mouse embryonic stem cells. RNA.

[10] Yamaguchi A, Abe M (2012). Regulation of reproductive development by non-coding RNA in Arabidopsis: to flower or not to flower. J Plant Res.

[11] Francozorrilla JM, Valli A, Todesco M, Mateos I, Puga MI, Rubiosomoza I, Leyva A, Weigel D, García JA, Pazares J (2007). Target mimicry provides a new mechanism for regulation of microRNA activity. Nat Genet.

[12] Muthusamy M, Uma S, Backiyarani S, Saraswathi MS (2015). Genome-wide screening for novel, drought stress-responsive long non-coding RNAs in drought-stressed leaf transcriptome of drought-tolerant and -susceptible banana ( Musa spp) cultivars using Illumina high-throughput sequencing. Plant Biotechnol Rep.

[13] Zhang X, Cao W, Wang Y, S Wang. Study of the progress on chemical constituents and pharmacological activities of Longan. Northwest Pharmaceutical J. 2012;27(5):493–6.

[14] Liang W, Chen W, Song R, Zhang F (2004). Advances in embryo development of longan. Subtrop Plant Sci.

[15] Lai Z. Study of Longan biotechnology. 1st ed. Fuzhou: Fujian Science and Technology Press; 2003.

[16] Lin Y, Lai Z (2013). Comparative Analysis Reveals Dynamic Changes in miRNAs and Their Targets and Expression during Somatic Embryogenesis in Longan (*Dimocarpus longan* Lour.). Plos One.

[17] Lin Y, Lai Z, Lin L, Lai R, Tian Q, Ye W, Zhang D, Yang M, Chen Y, Zhang Z (2015). Endogenous target mimics, microRNA167, and its targets ARF6 and ARF8 during somatic embryo development in *Dimocarpus longan* Lour. Mol Breed.

[18] Lin Y, Lin L, Lai R, Liu W, Chen Y, Zhang Z, XuHan X, Lai Z (2015). MicroRNA390-directed TAS3 cleavage leads to the production of tasiRNA-ARF3/4 during somatic embryogenesis in Dimocarpus longan Lour. Front Plant Sci.

[19] Lin Y, Lai Z, Tian Q, Lin L, Lai R, Yang M, Zhang D, Chen Y, Zhang Z (2015). Endogenous target mimics down-regulate miR160 mediation of ARF10, −16, and −17 cleavage during somatic embryogenesis in Dimocarpus longan Lour. Front Plant Sci.

[20] Chen X, Zeng Y, Wang J, Chen X, Xiaoping XU (2017). Evolutionary characteristics of miR159 gene family in *Dimocarpus longan* Lour., and their spatial and temporal expression. Chin J App Environ Biol.

[21] Zeng Y, Lin Y, Cui T, Chen X, Xiaoping XU (2017). Evolutionary characterization and Expresion of miR171 family in Dimocarpus longan Lour. Acta Botan Boreali-Occiden Sin.

[22] Lin YL, Lai ZX (2013). Evaluation of suitable reference genes for normalization of microRNA expression by real-time reverse transcription PCR analysis during longan somatic embryogenesis. Plant Physiol Biochem.

[23] Lai Z, Lin Y (2013). Analysis of the global transcriptome of longan (*Dimocarpus longan* Lour.) embryogenic callus using Illumina paired-end sequencing. BMC Genomics.

[24] Lin Y, Min J, Lai R, Wu Z, Chen Y, Yu L, Cheng C, Jin Y, Tian Q, Liu Q (2017). Genome-wide sequencing of longan (Dimocarpus longan Lour.) provides insights into molecular basis of its polyphenol-rich characteristics. Gigascience.

[25] Chekanova JA (2015). Long non-coding RNAs and their functions in plants. Curr Opin Plant Biol.

[26] Pertea M, Kim D, Pertea G, Leek JT, Salzberg SL (2016). Transcript-level expression analysis of RNA-seq experiments with HISAT, StringTie, and Ballgown. Nat Protoc.

[27] Roberts A, Pimentel H, Trapnell C, Pachter L (2011). Identification of novel transcripts in annotated genomes using RNA-Seq. Bioinformatics.

[28] Wang Y, Crawford DR, Davies KJ (1996). adapt33, a novel oxidant-inducible RNA from hamster HA-1 cells. Arch Biochem Biophys.

[29] Wang Y, Davies KJ, Melendez JA, Crawford DR (2003). Characterization of adapt33, a stress-inducible riboregulator. Gene Expr.

[30] Kohtz JD, Fishell G (2004). Developmental regulation of EVF-1, a novel non-coding RNA transcribed upstream of the mouse Dlx6 gene. Gene Expr Patterns.

[31] Mitsuya K, Meguro M, Lee MP, Katoh M, Schulz TC, Kugoh H, Yoshida MA, Niikawa N, Feinberg AP, Oshimura M (1999). LIT1, an imprinted antisense RNA in the human KvLQT1 locus identified by screening for differentially expressed transcripts using monochromosomal hybrids. Hum Mol Genet.

[32] Williams GD, Chang RY, Brian DA (1999). A phylogenetically conserved hairpin-type 3′ untranslated region pseudoknot functions in coronavirus RNA replication. J Virol.

[33] Bricaire F (2004). Questions about the West Nile virus. Presse Med.

[34] Lever A, Gottlinger H, Haseltine W, Sodroski J (1989). Identification of a sequence required for efficient packaging of human immunodeficiency virus type 1 RNA into virions. J Virol.

[35] Wu H, Wang Z, Wang M, Wang X (2013). Widespread long noncoding RNAs as endogenous target mimics for MicroRNAs in plants. Plant Physiol.

[36] Nemhauser JL, Feldman LJ, Zambryski PC (2000). Auxin and ETTIN in Arabidopsis gynoecium morphogenesis. Development.

[37] Hardtke C, Ckurshumova W, Vidaurre D, Singh S, Stamatiou G, Tiwari S, Hagen G, Guilfoyle T, Berleth T (2004). Overlapping and non-redundant functions of the Arabidopsis auxin response factors MONOPTEROS and NONPHOTOTROPIC HYPOCOTYL 4. Development.

[38] Xu Z, Yan Y, Qian L, Gong Z (2017). Long non-coding RNAs act as regulators of cell autophagy in diseases (review). Oncol Rep.

[39] Heo JB, Sung S (2011). Vernalization-mediated epigenetic silencing by a long intronic noncoding RNA. Science.

[40] Wasaki J, Yonetani RT, Kai M, Osaki M (2003). Expression of the OsPI1 gene, cloned from rice roots using cDNA microarray, rapidly responds to phosphorus status. New Phytol.

[41] Hao Z, Fan C, Cheng T, Su Y, Wei Q, Li G (2015). Genome-wide identification, characterization and evolutionary analysis of long intergenic noncoding RNAs in cucumber. PLoS One.

[42] Flórez-Zapata NMV, Reyes-Valdés MH, Martínez O (2016). Long non-coding RNAs are major contributors to transcriptome changes in sunflower meiocytes with different recombination rates. BMC Genomics.

[43] Joshi RK, Megha S, Basu U, Rahman MH, Kav NN (2016). Genome wide identification and functional prediction of long non-coding RNAs responsive to Sclerotinia sclerotiorum infection in Brassica napus. PLoS One.

[44] Kornienko AE, Guenzl PM, Barlow DP, Pauler FM (2013). Gene regulation by the act of long non-coding RNA transcription. BMC Biol.

[45] Knauss JL, Sun T (2013). Regulatory mechanisms of long noncoding RNAs in vertebrate central nervous system development and function. Neuroscience.

[46] Zhang L, Wang M, Li N, Wang H, Qiu P, Pei L, Xu Z, Wang T, Gao E, Liu J (2017). Long noncoding RNAs involve in resistance to Verticillium dahliae, a fungal disease in cotton. Plant Biotechnol J.

[47] Ge W, Zhang Y, Cheng Z, Hou D, Li X, Gao J (2017). Main regulatory pathways, key genes and microRNAs involved in flower formation and development of moso bamboo (Phyllostachys edulis). Plant Biotechnol J.

[48] Li M, Liang Z, He S, Zeng Y, Jing Y, Fang W, Wu K, Wang G, Ning X, Wang L (2017). Genome-wide identification of leaf abscission associated microRNAs in sugarcane (*Saccharum officinarum* L.). BMC Genomics.

[49] Cui K, Xing G, Zhou G, Liu X, Wang Y (2000). The induced and regulatory effects of plant hormones in somatic embryogenesis. Hereditas.

[50] Hadrami IE, Carron MP, D'Auzac J (1991). Influence of exogenous hormones on somatic embryogenesis in Hevea brasiliensis. Ann Bot.

[51] Zhu W, Mo R, Li K (2010). The Effects of Plant Hormones on the Somatic Embryogenesis and Plant Regeneration of Cassava. Chin J Trop Crops.

[52] Szemenyei H, Hannon M, Long JA (2008). TOPLESS mediates auxin-dependent transcriptional repression during Arabidopsis embryogenesis. Science.

[53] Lai R, Zhong C, Lin Y, Lai Z (2016). Cloning and expression analysis of auxin receptor gene TIR1 from Dimocarpus longan Lour. Chin J Trop Crops.

[54] Cohen DE, Davidow LS, Erwin JA, Xu N, Warshawsky D, Lee JT (2007). The DXPas34 repeat regulates random and imprinted X inactivation. Dev Cell.

[55] Lefevre P, Witham J, Lacroix CE, Cockerill PN, Bonifer C (2008). The LPS-induced transcriptional upregulation of the chicken lysozyme locus involves CTCF eviction and noncoding RNA transcription. Mol Cell.

[56] Faghihi MA, Modarresi F, Khalil AM, Wood DE, Sahagan BG, Morgan TE, Finch CE, St RLG, Kenny PJ, Wahlestedt C (2008). Expression of a noncoding RNA is elevated in Alzheimer’s disease and drives rapid feed-forward regulation of β-secretase expression. Nat Med.

[57] Huang B, Song JH, Cheng Y, Abraham JM, Ibrahim S, Sun Z, Ke X, Meltzer SJ (2016). Long non-coding antisense RNA KRT7-AS is activated in gastric cancers and supports cancer cell progression by increasing KRT7 expression. Oncogene.

[58] Aukerman MJ, Sakai H (2003). Regulation of flowering time and floral organ identity by a MicroRNA and its APETALA2-like target genes. Plant Cell.

[59] Schmid M, Uhlenhaut NH, Godard F, Demar M, Bressan R, Weigel D, Lohmann JU (2003). Dissection of floral induction pathways using global expression analysis. Development.

[60] Zhang J, Zhang S, Han S, Wu T, Li X, Li W, Qi L (2012). Genome-wide identification of microRNAs in larch and stage-specific modulation of 11 conserved microRNAs and their targets during somatic embryogenesis. Planta.

[61] Zhang J, Xue B, Gai M, Song S, Jia N, Sun H (2017). Small RNA and transcriptome sequencing reveal a potential miRNA-mediated interaction network that functions during somatic embryogenesis in Lilium pumilum DC. Fisch. Front Plant Sci.

[62] Wang S, Wu K, Yuan Q, Liu X, Liu Z, Lin X, Zeng R, Zhu H, Dong G, Qian Q (2012). Control of grain size, shape and quality by OsSPL16 in rice. Nat Genet.

[63] Nag A, King S, Jack T (2009). miR319a targeting of TCP4 is critical for petal growth and development in Arabidopsis. Proc Natl Acad Sci U S A.

[64] Zhou M, Li D, Li Z, Hu Q, Yang C, Zhu L, Luo H (2014). Constitutive expression of a miR319 gene alters plant development and enhances salt and drought tolerance in transgenic creeping Bentgrass. Plant Signal Behav.

[65] Lai Z, Pan L, Chen Z. Establishment and maintenance of longan embryogenic cell lines. J Fujian Agric For Univ. 1997;(2):160–7.

[66] Kim D, Langmead B, Salzberg SL (2015). HISAT: a fast spliced aligner with low memory requirements. Nat Methods.

[67] Kong L, Zhang Y, Ye ZQ, Liu XQ, Zhao SQ, Wei L, Gao G (2007). CPC: assess the protein-coding potential of transcripts using sequence features and support vector machine. Nucleic Acids Res.

[68] Sun L, Luo H, Bu D, Zhao G, Yu K, Zhang C, Liu Y, Chen R, Zhao Y (2013). Utilizing sequence intrinsic composition to classify protein-coding and long non-coding transcripts. Nucleic Acids Res.

[69] Finn RD, Coggill P, Eberhardt RY, Eddy SR, Mistry J, Mitchell AL, Potter SC, Punta M, Qureshi M, Sangradorvegas A (2016). The Pfam protein families database: towards a more sustainable future. Nucleic Acids Res.

[70] Audic S, Claverie JM (1997). The significance of digital gene expression profiles. Genome Res.

[71] Nawrocki EP, Kolbe DL, Eddy SR (2009). Infernal 1.0: inference of RNA alignments. Bioinformatics.

[72] Futschik ME, Carlisle B (2008). Noise-robust soft clustering of gene expression time-course data. J Bioinform Comput Biol.

[73] Kumar L, Futschik ME (2007). Mfuzz: a software package for soft clustering of microarray data. Bioinformation.

[74] Jia H, Osak M, Bogu GK, Stanton LW, Johnson R, Lipovich L (2010). Genome-wide computational identification and manual annotation of human long noncoding RNA genes. RNA.

[75] Tafer H, Hofacker IL (2008). RNAplex: a fast tool for RNA-RNA interaction search. Bioinformatics.

[76] Lobo I (2012). Basic local alignment search tool (BLAST). J Mol Biol.

[77] Buchfink B, Xie C, Huson DH (2015). Fast and sensitive protein alignment using DIAMOND. Nat Methods.

[78] Conesa A, Götz S, García-Gómez JM, Terol J, Talón M, Robles M (2005). Blast2GO: a universal tool for annotation, visualization and analysis in functional genomics research. Bioinformatics.

[79] Quevillon E, Silventoinen V, Pillai S, Harte N, Mulder N, Apweiler R, Lopez R (2005). InterProScan: protein domains identifier. Nucleic Acids Res.

[80] Wilusz JE, Sunwoo H, Spector DL (2009). Long noncoding RNAs: functional surprises from the RNA world. Genes Dev.

[81] Griffithsjones S, Grocock RJ, Van Dongen S, Bateman A, Enright AJ (2006). miRBase: microRNA sequences, targets and gene nomenclature. Nucleic Acids Res.

[82] Dai X, Zhao PX (2011). psRNATarget: a plant small RNA target analysis server. Nucleic Acids Res.

[83] Bonnet E, He Y, Billiau K, Van YDP (2010). TAPIR, a web server for the prediction of plant microRNA targets, including target mimics. Bioinformatics.

[84] Lin Y, Lai R, Lai Z, Cheng C, Liu S (2016). Method for enhancing and carrying out suppression expression on activity of miRNA in embryogenic callus of longan..

