# Supplementary material for: Genome-wide identification and characterization of long non-coding RNAs involved in the early somatic embryogenesis in Dimocarpus longan Lour
Source: BMC Genomics. 2018 Nov 6;19:805. doi: 10.1186/s12864-018-5158-z (PMC6219066; doi:10.1186/s12864-018-5158-z)
Supplement: Supplementary file 5 — KEGG annotation analysis of potential target genes of lncRNAs. Functional categorization of potential target genes of lncRNAs based on the biological process category of the Kyoto Encyclopedia of Genes and Genomes (KEGG). (PDF 204 kb) [file 12864_2018_5158_MOESM5_ESM.pdf]

Fig.S1 KEGG annotation analysis of potential target genes of lncRNAs. Functional categorization of potential target genes of lncRNAs based on the biological process category of the Kyoto Encyclopedia of Genes and Genomes (KEGG).

EC-VS-GE

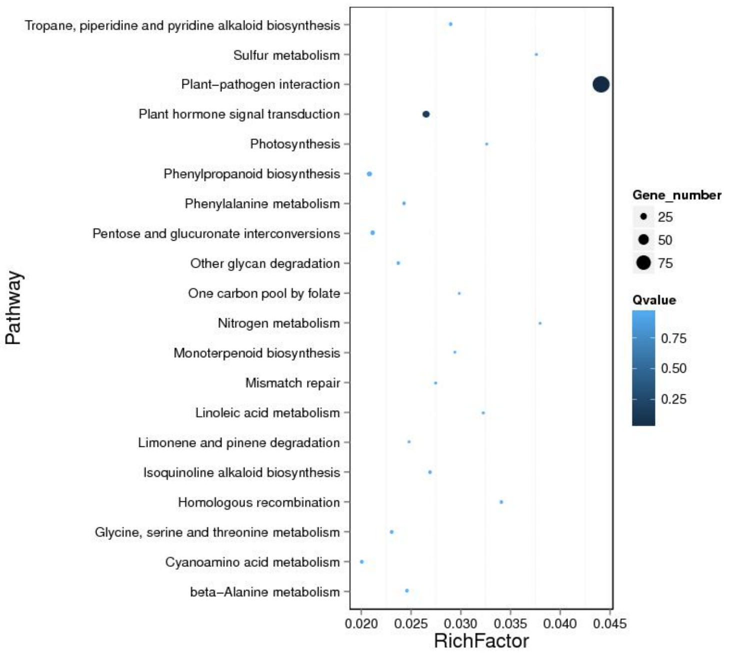

EC-VS-ICpEC

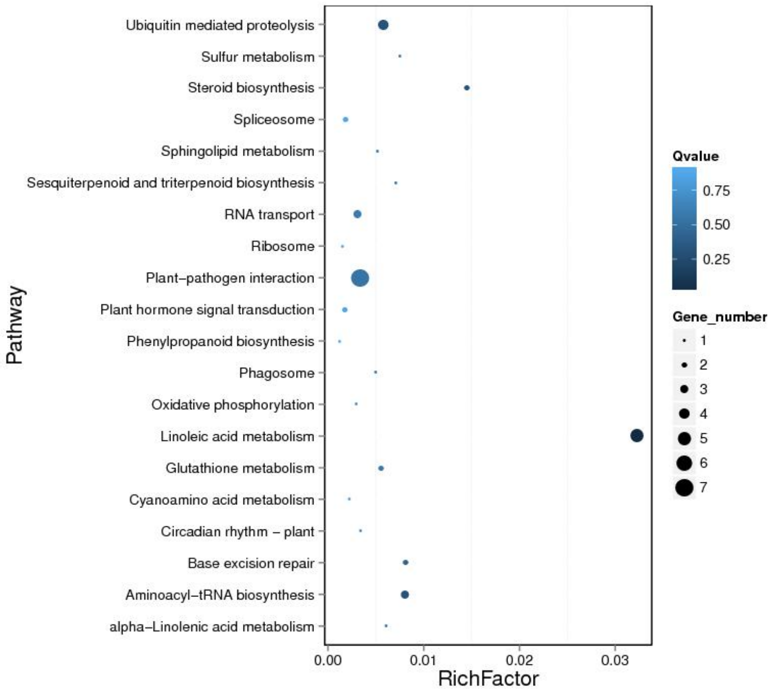

ICpEC-VS-GE

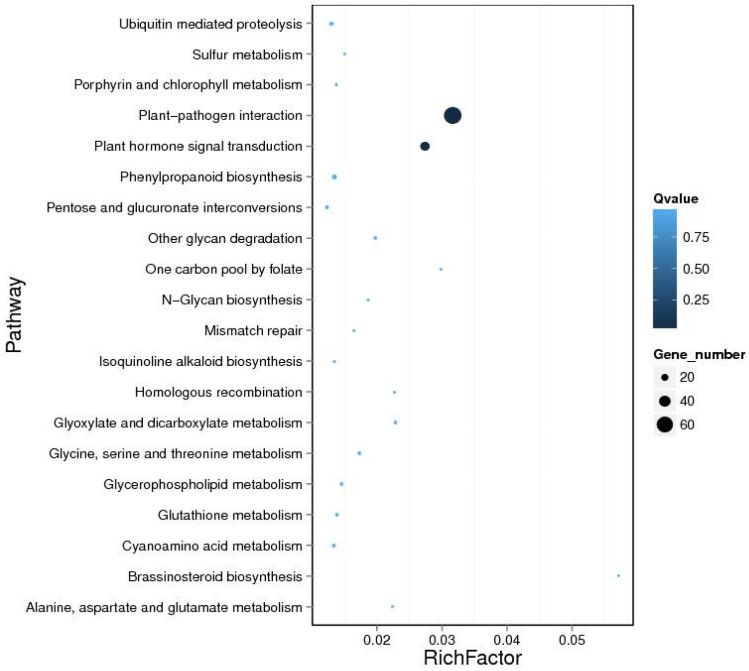
Ubiquitin mediated proteolysis
Sulfur metabolism
Porphyrin and chlorophyll metabolism
Plant−pathogen interaction
Plant hormone signal transduction
Phenylpropanoid biosynthesis
Pentose and glucuronate interconversions
Other glycan degradation
One carbon pool by folate
N−Glycan biosynthesis
Mismatch repair
Isoquinoline alkaloid biosynthesis
Homologous recombination
Glyoxylate and dicarboxylate metabolism
Glycine, serine and threonine metabolism
Glycerophospholipid metabolism
Glutathione metabolism
Cyanoamino acid metabolism
Brassinosteroid biosynthesis
Alanine, aspartate and glutamate metabolism
Pathway
0.02
0.03
0.04
0.05
RichFactor
Qvalue
0.75
0.50
0.25
Gene_number
20
40
60
