# Supplementary material for: Genome-wide identification and characterization of long non-coding RNAs involved in the early somatic embryogenesis in Dimocarpus longan Lour
Source: BMC Genomics. 2018 Nov 6;19:805. doi: 10.1186/s12864-018-5158-z (PMC6219066; doi:10.1186/s12864-018-5158-z)
Supplement: Supplementary file 7 — qPCR validation of five miRNAs involved in “auxin response” regulation of lncRNAs. Dlo-miR172a was used as a reference gene to normalize miRNA expression data; FSD was used to normalize lncRNAs and mRNAs. (PDF 406 kb) [file 12864_2018_5158_MOESM7_ESM.pdf]

Fig.S2 qPCR validation of five miRNAs involved in "auxin response" regulation of lncRNAs. Dlo-miR172a was used as a reference gene to normalize miRNA expression data; *FSD* was used to normalize lncRNAs and mRNAs.

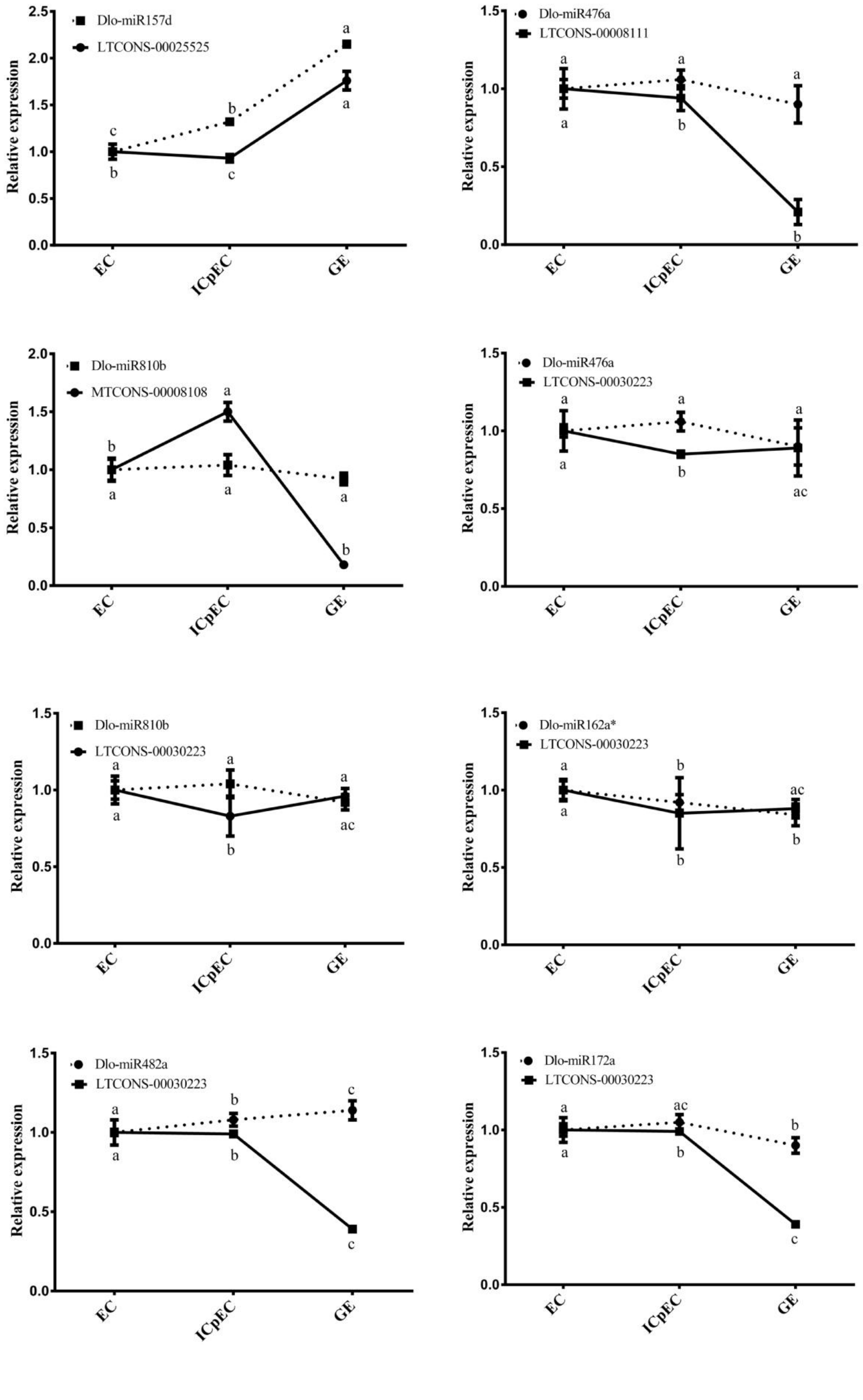

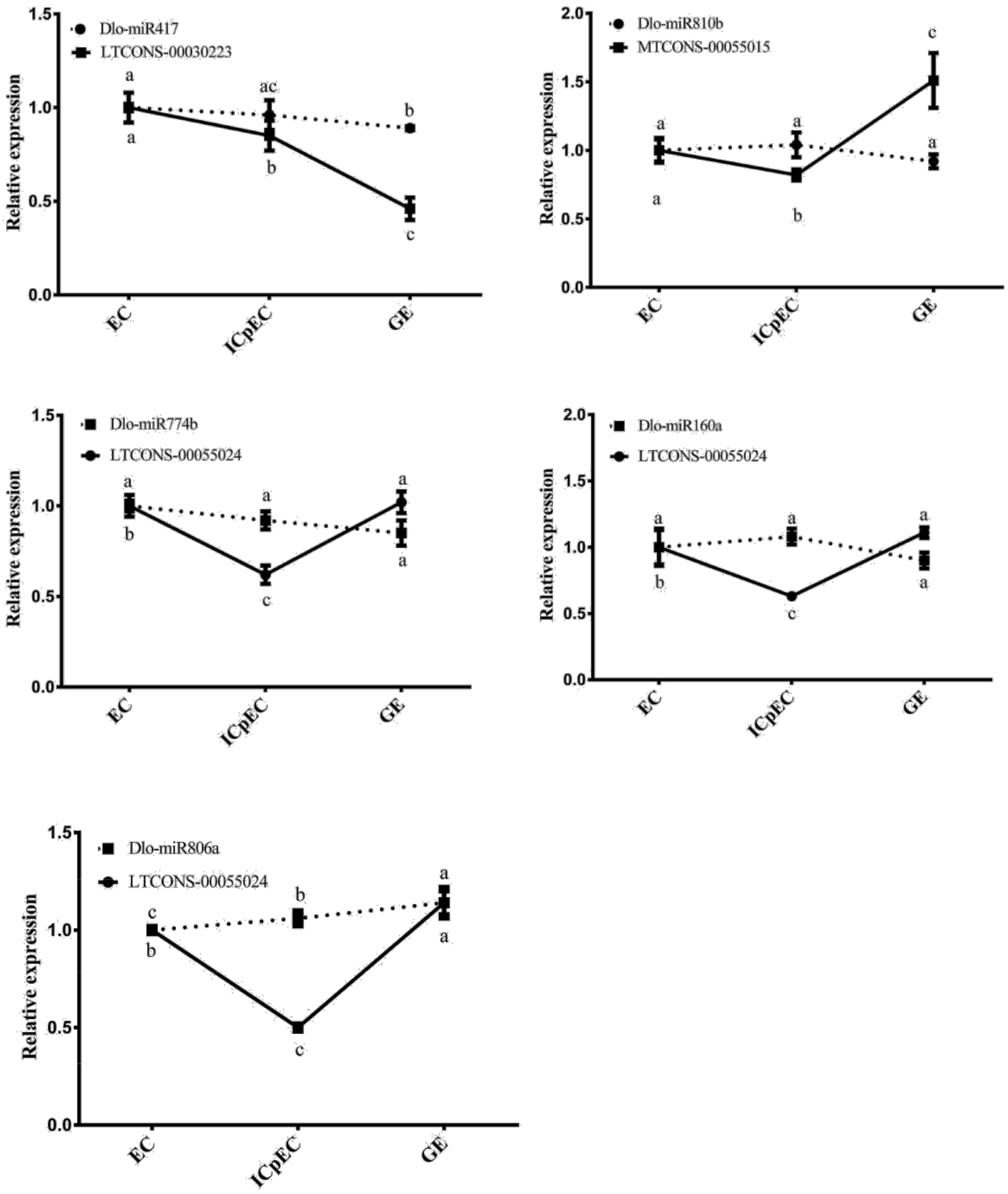
Dlo-miR417
LTCONS-00030223
Relative expression
EC
ICpEC
GE
Dlo-miR810b
MTCONS-00055015
Relative expression
EC
ICpEC
GE
Dlo-miR774b
LTCONS-00055024
Relative expression
EC
ICpEC
GE
Dlo-miR160a
LTCONS-00055024
Relative expression
EC
ICpEC
GE
Dlo-miR806a
LTCONS-00055024
Relative expression
EC
ICpEC
GE
