# Supplementary material for: Genome-wide identification and characterization of long non-coding RNAs involved in the early somatic embryogenesis in Dimocarpus longan Lour
Source: BMC Genomics. 2018 Nov 6;19:805. doi: 10.1186/s12864-018-5158-z (PMC6219066; doi:10.1186/s12864-018-5158-z)
Supplement: Supplementary file 8 — qPCR validation of the lncRNA-miRNA-mRNA relationships of the miR159a.1 and miR398a networks. (a) miR159a.1 network qPCR validation. Dlo-miR396a was used as a reference gene to normalize miRNA expression data; DlRan3A was used to normalize lncRNAs and mRNAs. (b) miR398a network qPCR validation. Dlo-miR164a was used as a reference gene to normalize miRNA expression data; EF-1α was used to normalize lncRNAs and mRNAs. (PDF 1266 kb) [file 12864_2018_5158_MOESM8_ESM.pdf]

Fig.S3 qPCR validation of the lncRNA-miRNA-mRNA relationships of the miR159a.1 and miR398a networks. (a) miR159a.1 network qPCR validation. Dlo-miR396a was used as a reference gene to normalize miRNA expression data; *DlRan3A* was used to normalize lncRNAs and mRNAs. (b) miR398a network qPCR validation. Dlo-miR164a was used as a reference gene to normalize miRNA expression data; *EF-1α* was used to normalize lncRNAs and mRNAs.

a

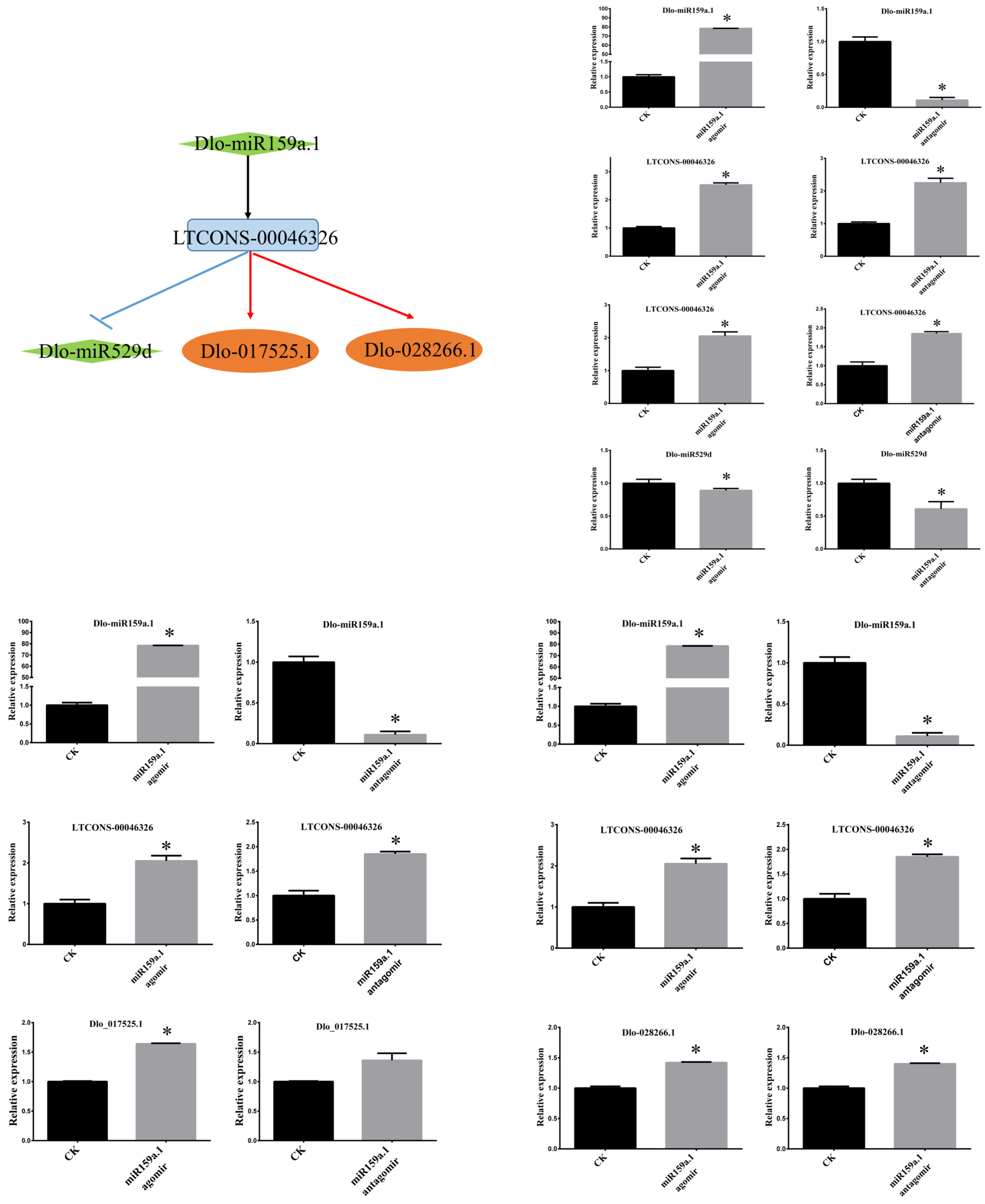

b

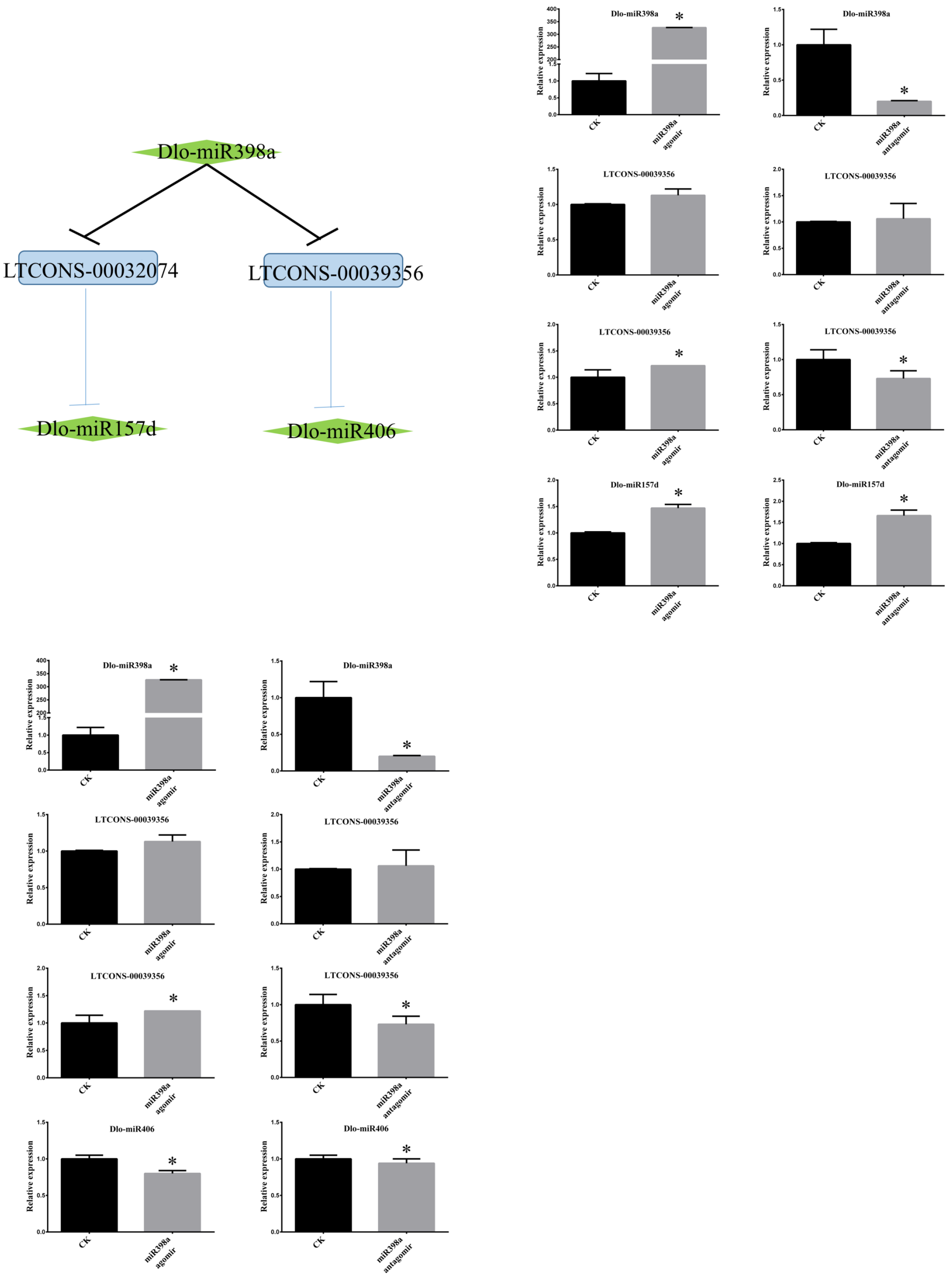
Dlo-miR398a
LTCONS-00032074
LTCONS-00039356
Dlo-miR157d
Dlo-miR406
Dlo-miR398a
LTCONS-00039356
Dlo-miR157d
Dlo-miR406
Relative expression
CK
miR398a agomir
miR398a antagomir
*
